# *Ixodes scapularis* nymph saliva protein blocks host inflammation and complement-mediated killing of Lyme disease agent, *Borrelia burgdorferi*

**DOI:** 10.3389/fcimb.2023.1253670

**Published:** 2023-10-26

**Authors:** Emily Bencosme-Cuevas, Tae Kwon Kim, Thu-Thuy Nguyen, Jacquie Berry, Jianrong Li, Leslie Garry Adams, Lindsey A. Smith, Syeda Areeha Batool, Daniel R. Swale, Stefan H. E. Kaufmann, Yava Jones-Hall, Albert Mulenga

**Affiliations:** ^1^ Department of Veterinary Pathobiology, School of Veterinary Medicine and Biomedical Sciences, Texas A&M University, College Station, TX, United States; ^2^ Department of Diagnostic Medicine/Pathobiology, College of Veterinary Medicine, Kansas State University, Manhattan, KS, United States; ^3^ Department of Veterinary Integrative Biosciences, School of Veterinary Medicine and Biomedical Sciences, Texas A&M University, College Station, TX, United States; ^4^ Department of Veterinary Physiology and Pharmacology, School of Veterinary Medicine and Biomedical Sciences, Texas A&M University, College Station, TX, United States; ^5^ Aiforia Technologies, Cambridge, MA, United States; ^6^ Emerging Pathogens Institute, University of Florida, Gainesville, FL, United States; ^7^ Hagler Institute for Advanced Study, Texas A&M University, College Station, TX, United States; ^8^ Max Planck Institute for Infection Biology, Berlin, Germany; ^9^ Max Planck Institute for Multidisciplinary Sciences, Göttingen, Germany

**Keywords:** *Borrelia burgdorferi*, *Ixodes scapularis*, inflammation, chymase, cathepsin G, complement system, membrane attack complex

## Abstract

Tick serine protease inhibitors (serpins) play crucial roles in tick feeding and pathogen transmission. We demonstrate that Ixodes scapularis (Ixs) nymph tick saliva serpin (S) 41 (IxsS41), secreted by Borrelia burgdorferi (Bb)-infected ticks at high abundance, is involved in regulating tick evasion of host innate immunity and promoting host colonization by Bb. Recombinant (r) proteins were expressed in Pichia pastoris, and substrate hydrolysis assays were used to determine. Ex vivo (complement and hemostasis function related) and in vivo (paw edema and effect on Bb colonization of C3H/HeN mice organs) assays were conducted to validate function. We demonstrate that rIxsS41 inhibits chymase and cathepsin G, pro-inflammatory proteases that are released by mast cells and neutrophils, the first immune cells at the tick feeding site. Importantly, stoichiometry of inhibition analysis revealed that 2.2 and 2.8 molecules of rIxsS41 are needed to 100% inhibit 1 molecule of chymase and cathepsin G, respectively, suggesting that findings here are likely events at the tick feeding site. Furthermore, chymase-mediated paw edema, induced by the mast cell degranulator, compound 48/80 (C48/80), was blocked by rIxsS41. Likewise, rIxsS41 reduced membrane attack complex (MAC) deposition via the alternative and lectin complement activation pathways and dose-dependently protected Bb from complement killing. Additionally, co-inoculating C3H/HeN mice with Bb together with rIxsS41 or with a mixture (rIxsS41 and C48/80). Findings in this study suggest that IxsS41 markedly contributes to tick feeding and host colonization by Bb. Therefore, we conclude that IxsS41 is a potential candidate for an anti-tick vaccine to prevent transmission of the Lyme disease agent.

## 1 Introduction

The impact of ticks and tick-borne diseases (TBDs) on human and veterinary health was recognized as early as 1550 BC when tick fever was mentioned on an Egyptian papyrus scroll (Galun and Obenchain, 1982; Anderson, 2002). In the past two decades, the incidence of TBDs has more than doubled in the United States (Centers for Disease Control and Prevention, 2021), and as of 2023, 17 TBDs have been reported as cause for human morbidity by the Centers for Disease Control and Prevention (CDC) (Eisen et al., 2017). Among these TBDs, Lyme disease (LD) remains as the most reported to date (Centers for Disease Control and Prevention, 2019, January 13, 2021).

LD is caused by the spirochete-shaped bacteria *Borrelia burgdorferi* (Scarpa et al., 1994) and *Borrelia mayonii* (Pritt et al., 2016), and is the most common human vector borne disease (HVBD) in the United States. As of January 2022, insurance records reveal that more than 400,000 Americans have been diagnosed and treated for LD annually (Centers for Disease Control and Prevention January 13, 2021), and incidents have increased by more than 300% in northeastern states (Kugeler et al., 2015). Studies have shown that climate change has contributed to this increase, making more areas in North America suitable for the survival of *Ixodes scapularis*, the vector host for the causative agent of LD (Dumic and Severnini, 2018; Sonenshine, 2018; Centers for Disease Control and Prevention).

Currently, there are no effective vaccines in the market to prevent *B. burgdorferi* infections in humans (Nigrovic and Thompson, 2007), and prevention of LD relies on preventing infectious tick bites. Reducing vector tick populations in nature is expected to result in fewer infectious tick bites and LD cases. Despite a plethora of methods including acaricides, repellents, and proper personal protection (Przygodzka et al., 2019), LD cases have continued to rise (Abbas et al., 2014; Centers for Disease Control and Prevention May 5 2021; Rosario-Cruz et al., 2009). Tick antigen-based vaccines have shown promise as an alternative to prevent LD (Ali et al., 2020). This is supported by findings that indicate that acquired immunity to tick saliva proteins by repeatedly infested animals confers potent protection against both tick feeding and infection with tick-borne pathogens (TBPs) including *B. burgdorferi* (Wikel et al., 1997; Nazario et al., 1998). With the goal of identifying key tick saliva proteins that can be targeted for anti-tick feeding vaccine development, our laboratory and others have identified tick saliva proteins by LC-MS/MS proteomics, which are injected into animals by adult and nymph (uninfected and *B. burgdorferi* infected) *I. scapularis* (Tirloni et al., 2017; Kim et al., 2016a; Radulović et al., 2014; Kim et al., 2021), adult *Amblyomma americanum* (Porter et al., 2015), cattle ticks, *Rhipicephalus microplus* (Tirloni et al., 2014), the Asian longhorned tick *Haemaphysalis longicornis* (Tirloni et al., 2015), and *Dermacentor andersoni* (Mudenda et al., 2014). However, identifying these tick saliva proteins is only the first step to fully understanding their roles in tick feeding physiology. The next step of the research is to understand the functional roles of tick saliva proteins in regulating tick feeding and TBPs.

The tick feeding style of disrupting host skin tissue using their chelicerae and then sip blood that bleeds into the feeding site triggers innate immune defense mechanisms including inflammation, hemostasis (platelet aggregation and blood clotting), complement activation, and other tissue repair responses. Since several components of the innate immune system are part of serine protease-mediated cascades that are controlled by serpins (Gettins, 2002; Huntington, 2006; Rau et al., 2007; Meekins et al., 2017), ticks were hypothesized to utilize these proteins to regulate feeding and thus represent attractive anti-tick vaccine targets (Chmelař et al., 2017).

Since the thought-provoking manuscript (Muleng et al., 2001), expression of serpins has been confirmed in several tick species (Kim et al., 2016a; Kim et al., 2020b), as the largest class of protease inhibitors that ticks inject into the host during feeding (Chmelař et al., 2017; Kim et al., 2021). Several studies have confirmed that some of the tick saliva serpins are inhibitors of serine protease effectors of the innate immune system (Chalaire et al., 2011; Mulenga et al., 2013; Ibelli et al., 2014; Kim et al., 2015; Kim et al., 2016b; Chmelař et al., 2017; Bakshi et al., 2018; Bakshi et al., 2019; Tirloni et al., 2019; Kim et al., 2020a), indicating their roles in tick evasion of innate immunity. The objective of this study was to characterize a tick saliva serine protease inhibitor (serpin) highly secreted by *I. scapularis* nymphs that are infected with *B. burgdorferi* (Kim et al., 2021).

We have functionally characterized *I. scapularis* (*Ixs*) tick serpin (S) 41 (*Ixs*S41) (Mulenga et al., 2009) that is abundantly secreted by *B. burgdorferi-*infected nymphs (Kim et al., 2021). We show that *Ixs*S41 is not part of a redundant system but is highly conserved across *Ixodes* spp. and that it is an anti-inflammatory protein that likely influences *B. burgdorferi* colonization of the host.

## 2 Materials and methods

### 2.1 Ethics statement

All experiments were done according to the animal use protocol approved by Texas A&M University Institutional Animal Care and Use Committee (IACUC) (AUP 2018-001 and 2020-0089) that meets all federal requirements, as defined in the Animal Welfare Act (AWA), the Public Health Service Policy (PHS), and the Humane Care and Use of Laboratory Animals.

### 2.2 Cloning, sequencing, and phylogeny of *Ixodes scapularis* serpin (S) EEC14235.1

The *Ixs*S41(accession #EEC14235.1 and XP_002402368.4) sequence was obtained from GenBank. Cognizant of the fact that there are some errors in sequences that have been deposited in GenBank, nested 3′ prime rapid amplification of cDNA ends (RACE) primers (Forward: 5′GGTCGGTGGCGTTGTCTCGGGAGGTA3′ [used with the company provided Universal Primer A Mix (UPM)] and Forward Nested: 5′ATGAAGACTCTGGCAGCATTCCTGTC3′) [used with the company provided Universal Primer Short] were designed based on the EEC14235 sequence in GenBank. We designed long PCR primers to match the high annealing temperatures of universal primers in the kit. The RACE template was synthesized from available fed adult ticks using the SMARTer Clontech 3′ RACE kit according to instructions by the manufacturer (Takara Bio, San Jose, CA, USA). The primary and nested *IxsS*41 specific forward primers were used with universal primers in the Clontech kit. The nested PCR product was cloned into pGEMT cloning vector (Promega Corporation, Madison, WI, USA) and processed for DNA Sanger sequencing using T7 and SP6 promoter primers.

Using the sequence analysis application MacVector (North Carolina, USA), the translated amino acid sequence of *Ixs*S41 that was cloned in this study was compared to EEC14235.1 and XP_002402368.4 amino acid sequences from GenBank. To gain insight into the relationship of *Ixs*S41 with other tick serpins in GenBank, 22 tick proteins that showed at least 50% amino acid identity to *Ixs*S41 were downloaded alongside an outlier *Homo sapiens* alpha-1-antitrypsin precursor (NP_001121178.1). These serpin sequences were used to construct the phylogeny tree by the neighbor-joining method in MacVector with parameters set to detect absolute differences and bootstrap values at 1,000 replications. The 22 serpin sequences that were downloaded include *Dermacentor andersoni* (*n* = 1), *Dermacentor silvarum* (*n* = 1), *Ixodes persulcatus* (*n* = 2), *Ixodes ricinus* (*n* = 3), *Ixodes scapularis* (*n* = 13), *Rhipicephalus microplus* (*n* = 1), and *Rhipicephalus sanguineus* (*n* = 1). Additionally, N-glycosylation and O-glycosylation site prediction servers, NetnGlyc-1.0, and NetOGlyc-4.0 (DTU Health Technology, Lyngby, Denmark) were used to predict N- and O-linked glycosylation sites of r*Ixs*S41.

### 2.3 Expression and mass spectrometry analysis of recombinant (r) *Ixs*S41

The expression plasmid for mature r*Ixs*S41 containing a hexa-histidine tag at the C-terminal end was custom synthesized and cloned into the pPICZαA plasmid (Biomatik, Cambridge, Canada). Expression in *Pichia pastoris* X-33 cells and affinity purification of r*Ixs*S41 were done as published (Tirloni et al., 2019; Kim et al., 2020a). The pPICZαA plasmid and X-33 cell expression system secretes the recombinant protein into culture media, and the expressed r*Ixs*S41 was precipitated by ammonium sulfate saturation. For affinity purification, precipitates were resuspended and dialyzed against column binding buffer (20 mM Tris-HCl, 50 mM NaCl, and 5 mM imidazole, pH 7.4) and the expression of r*Ixs*S41 was confirmed by Western blot using mouse anti-hexa-histidine tag 1:5,000 diluted (GenScript Biotech, Piscataway, NJ, USA) and silver-stained following manufacturer instructions (Pierce Silver Stain Kit, ThermoFisher Scientific, Waltham, MA). The r*Ixs*S41 was subsequently purified under native conditions using a HiTrap Chelating HP column (Cytiva Life Sciences, Marlborough, MA, USA) and Tris-HCl-Imidazole buffer as published (Tirloni et al., 2019; Kim et al., 2020a). Buffer exchange into Tris-HCl buffer (20 mM Tris-HCl and 150 mM NaCl, pH 7.4) and volume reduction of affinity-purified r*Ixs*S41 were done with a Pall Corporation Microsep Advance Centrifugal Device (30-kDa molecular weight cutoff) (Pall Corporation, Port Washington, NY, USA). The r*Ixs*S41 concentration was quantified using the Pierce BCA Protein Assay Kit (ThermoFisher Scientific).

Preliminary SDS-PAGE and silver staining analysis revealed that r*Ixs*S41 was migrating as a diffuse band, suggesting that it was glycosylated. Thus, to confirm this, affinity-purified r*Ixs*S41 was subjected to deglycosylation using NEB Protein Deglycosylation Mix II, which removes both N- and O-linked glycans under both denaturing and non-denaturing conditions following the company-provided protocol (NEB, Inswic, MA, USA).

Preliminary silver staining analysis of deglycosylated r*Ixs*S41 (under denaturing and non-denaturing conditions) showed multiple bands. To confirm the identity of multiple bands, both deglycosylated and glycosylated versions of r*Ixs*S41 (7 µg each) were resolved on a 7.5% SDS-PAGE gel and silver stained using the Pierce Silver Stain for Mass Spectrometry (ThermoFisher Scientific). Single bands were then excised and submitted to the University of Florida Interdisciplinary Center for Biotechnology Research (ICBR) Proteomics & Mass Spectrometry Facility for LC-MS/MS analysis using the in-gel digestion approach (45 mM dithiothreitol [DTT], alkylated with 100 mM chloroacetamide [2-CAA], and trypsin-Lys-C). Using the Mascot program (Matrix Science, London, United Kingdom; version 2.7.0), tandem mass spectra were searched against the database of *Pichia pastoris* proteins (16,167 entries) and the amino acid sequence of *Ixs*S41. Protein identifications were accepted if they could be established at greater than 95.0% probability and contained at least 2 identified peptides. Protein probabilities were assigned by the Protein Prophet algorithm (Nesvizhskii et al., 2003) and those that contained similar peptides and could not be differentiated based on MS/MS analysis alone were grouped to satisfy the principles of parsimony.

### 2.4 Western blotting and ELISA analyses to determine immunogenicity of r*Ixs*S41

To determine if native *Ixs*S41 elicits an antibody response in rabbits, an empirically optimized r*Ixs*S41 amount (2 µg) was resolved on a 10% SDS-PAGE gel and transferred onto an Immobilon-P PVDF Membrane (Sigma-Aldrich, Burlington, MA, USA). Sera of rabbits that were repeatedly infested (or fed on twice) with uninfected and *B. burgdorferi-*infected *I. scapularis* nymph ticks (Ibelli et al., 2014; Bakshi et al., 2018; Kim et al., 2021) diluted at 1:200 and 1:1,000 were used to probe PVDF membranes at 4°C overnight. The membranes were thoroughly washed in phosphate buffered saline (PBS)-Tween 20, 0.05% (T), and 1:2,000 diluted goat anti-rabbit IgG HRP-conjugated antibody (SouthernBiotech, Birmingham, AL, USA) was used as secondary antibody for 1 h at room temperature. To detect the positive signal, washed PVDF membranes were incubated with the SuperSignal West Dura Extended Duration Substrate (ThermoFisher Scientific) for 10 min at room temperature and signal was detected using ChemiDocXRS+ imager (Bio-Rad, Hercules, CA, USA).

For ELISA analysis, high-binding 96-well plates (Corning, NY, USA) were coated with increasing amounts of r*Ixs*S41 (0.12–1.00 µg) in triplicates overnight at 4°C. The following day, the wells were washed three times with PBS-T and blocked with 5% skim milk powder in PBS-T for 1 h at room temperature. Subsequently, wells were incubated overnight at 4°C with serially diluted rabbit serum 1:200, 1:400, 1:600, 1:800, and 1:1,000. Preliminary Western blotting analysis revealed that binding intensity of pre-immune sera was diminished in diluted antibodies. Thus, pre-immune sera was only tested at 1:200. Following washing, wells were incubated with 1:2,000 diluted goat anti-rabbit IgG HRP-conjugated antibody (SouthernBiotech) for 1 h at room temperature. Following another round of washing, the 1-Step Ultra TMB-ELISA substrate (ThermoFisher Scientific) and 2N sulfuric acid (VWR, Radnor, PA, USA) were added and *A_450nm_* was recorded with the Synergy H1 plate reader (BioTek Instruments Inc., Winooski, VA, USA).

### 2.5 Inhibitory activity profiling of r*Ixs*S41 against mammalian serine proteases

The inhibitory activity of r*Ixs*S41 was profiled against 18 mammalian serine proteases involved in host defense mechanisms against tick feeding. Excess amount of r*Ixs*S41 (1 µM in 20 mM Tris-HCl, 50 mM NaCl, and Tween 0.1%, pH 7.4) was pre-incubated with each serine protease for 15 min at 37°C followed by the addition of its respective substrate (200 µM). Hydrolysis of the substrate was measured every 11 s for 15 min at 30°C at *A*_405nm_ using the Synergy H1 plate reader (BioTek Instruments Inc.) as previously described (Horvath et al., 2011; Kim et al., 2020a) and data were analyzed with published formulas in Horvath et al. (2011).

Additionally, the *in silico* protein-to-protein server PSOPIA (prediction server of protein-to-protein interactions; https://mizuguchilab.org/PSOPIA/) (Murakami and Mizuguchi, 2014) was used to predict interactions between *Ixs*S41 and the 18 mammalian serine proteases. Interactions with an Averaged One-Dependence Estimators (AODE) of more than 0.75 were considered significant (Murakami and Mizuguchi, 2014).

### 2.6 Stoichiometry of inhibition and rate of inhibitory reaction analysis

The stoichiometry of inhibition (SI) of r*Ixs*S41 was determined for proteases whose enzymatic activity was inhibited by more than 80%. Various concentrations of r*Ixs*S41 were incubated with a constant concentration of each protease for 1 h at 37°C. Colorimetric substrates were used to measure residual enzymatic activity and data were plotted as previously described (Horvath et al., 2011; Kim et al., 2015; Tirloni et al., 2019; Kim et al., 2020a). Forthwith, the second-order rate constant (*k*_a_) for the inhibition of chymase and cathepsin G (proteases whose SI values were below 7) by r*Ixs*S41 was determined using the discontinuous method (Horvath et al., 2011). Increasing amounts of r*Ixs*S41 (25–300 nM for chymase or 100–1600 nM for cathepsin G) were pre-incubated for 0–15 min at 37°C with constant amounts of chymase or cathepsin G to obtain the residual enzymatic activity. The *k_obs_* (pseudo-first order constant) was obtained from the slope of a semi-log plot of the residual chymase or cathepsin G activity against incubation time. The best-fit line of *k_obs_* values was plotted against different amounts of r*Ixs*S41, thus producing the second-order rate constant *k*_a_ (Horvath et al., 2011; Kim et al., 2015; Tirloni et al., 2019; Kim et al., 2020a).

### 2.7 r*Ixs*S41–protease complex formation

Complex formation between r*Ixs*S41 and targeted proteases whose enzymatic activity was inhibited by more than 80% and an SI value below 7 was determined. Chymase and Cathepsin G (0.1 µg) were incubated with r*Ixs*S41 at varying molar ratios (protease-to-r*Ixs*S41 of 10–0.625:1) for 1 h at 37°C. The reaction was stopped by adding denaturing SDS-PAGE reducing sample buffer and heat denaturation at 95°C for 5 min. Denatured samples were resolved on a 10% SDS-PAGE gel and visualized by silver staining using the Pierce Silver Stain Kit (ThermoFisher Scientific) (Tirloni et al., 2019). The r*Ixs*S41 and protease complex was detected at a high molecular weight approximately equal to the sum of the molecular size of r*Ixs*S41 and the target proteases.

### 2.8 Effects of r*Ixs*S41 on deposition of the complement membrane attack complex

The effect of r*Ixs*S41 on deposition of the membrane attack complex (MAC) via each of the three activation pathways of complement—Classic, Alternative, and Mannose Binding Lectin (MBL) systems—was tested using the WiesLab Complement System kit (Svar Life Science AB, Malmö, Sweden). The kit quantifies C5b-9 neoantigen formation with the use of specific alkaline phosphatase labeled antibodies. Affinity-purified r*Ixs*S41 (4 µM) was pre-incubated with human serum, provided by the company (positive control), for 30 min at 37°C. Thereafter, treatments (negative control [NC, containing human serum], positive control [PC, containing human serum that was originally freeze dried], and r*Ixs*S41 + PC) were added, in duplicate, to their respective wells and incubated at 37°C for 60 min, followed by the addition of conjugate and substrate. The *A*_405nm_ was obtained using the Synergy H1 plate reader following manufacturer instructions and MAC deposition calculated using the following formula: 


$$\frac{\left(\text{Sample}-\text{NC}\right)}{\begin{array}{c} \left(\text{PC}-\text{NC}\right) \end{array}}\times 100$$

Preliminary analysis revealed that r*Ixs*S41 reduced deposition of MAC via the MBL and alternative complement activation pathways. Thus, to gauge insight into possible r*Ixs*S41 targets in the complement system, the PSOPIA prediction server was utilized to evaluate interactions between *Ixs*S41 and proteases involved in the complement pathways (MASP1-3, C1r, C1s, C2, factor B, factor D, and factor I) as described above. Additionally, the interaction between r*Ixs*S41 and factor H was also evaluated as negative control given that factor H is not a protease.

### 2.9 Serum complement sensitivity assay

*B. burgdorferi* complement-resistant B314/BBK32 (pCD100) (Garcia et al., 2016) and complement-sensitive B314/pBBE22*luc* strains, kindly gifted by Dr. Jon T. Skare (TAMU College of Medicine), were cultured in BSK-II media at 32°C with 1% CO_2_ until mid-log phase. Thereafter, BSK-II media was used to dilute cultures to a concentration of 1 × 10^6^ spirochetes/mL. To investigate the effect of r*Ixs*S41 on *B. burgdorferi* viability throughout serum complement exposure, decreasing amounts (4, 2, and 1 µM) of r*Ixs*S41 were pre-incubated with normal human serum or heat-inactivated normal human serum (Complement Technology, Inc., Tyler, TX, USA) in a microtiter plate for 30 min at 37°C. Thereafter, 85 µL of either B314/BBK32 or B314/pBBE22*luc* diluted to 1 × 10^6^ spirochetes/mL was added to its respective well and incubated for 1.5 h at 32°C with shaking at 100 rpm. Spirochete survival was scored under dark field microscopy on 10 chosen fields 1.5 h after the start of incubation and every 30 min thereafter. We focused on identifying spirochetes that showed signs of (1) immobilization and (2) membrane damage (bacteriolysis), which were then recorded as not viable as previously described (Kurtenbach et al., 1998; Xie et al., 2019; Booth et al., 2022).

### 2.10 Paw edema assay

Prompted by data that showed r*Ixs*S41 inhibition of pro-inflammatory proteases, chymase, and cathepsin G, we sought to investigate r*Ixs*S41 anti-inflammatory effect *in vivo.* The paw edema assay was performed using C48/80 as the inflammation agonist on retired female BALB/c mice as described (Tirloni et al., 2019; Kim et al., 2020a). The experiment was completed twice, each involving 12 mice divided into four treatment groups: saline, C48/80 (1 µg), r*Ixs*S41 (25 µg), and mix (C48/80 [1 µg] and r*Ixs*S41 [25 µg]). The left hind paw of each mouse was carefully pre-marked to the same height and the initial volume was measured using a digital plethysmometer (Harvard Apparatus Inc.). The treatments were respectively administered in 20 µL total volume using a 31-gauge (0.25 mm) 15/64 in (6 mm) 3/10 cc (0.3 mL) U100 needle. Paw swelling (inflammation) was measured by volume displacement using the digital plethysmometer for 8 h: every 30 min for the first 2 h and every hour thereafter. Mice were then humanely euthanized by CO_2_ and cervical dislocation.

### 2.11 Histopathology and digital analysis

Three retired female BALB/c mice per treatment as described in the previous section (Paw Edema Assay) were injected with a 27-gauge needle on the left basal footpad. Given that the peak of inflammation during the paw edema assay took place 30–50 min after injection, mice were humanely euthanized as described before, 50 min after injection, and the skin injection site was immediately excised with a scalpel. The collected skin tissues were fixed in 4% paraformaldehyde for 13 h at room temperature. Post-fixation, the sections were thoroughly washed in 70% ethanol for submission to the Texas A&M School of Veterinary Medicine and Biomedical Sciences Core Histology Lab (RRID : SCR_022201) for routine processing and staining with hematoxylin & eosin (H&E) and toluidine blue (TB). The glass slides were scanned at 20× magnification using the Pannoramic Scan II by 3DHistec (Budapest, Hungary).

These scans were analyzed, in a blinded manner, by a board-certified pathologist, Dr. L. Garry Adams (VMBS), and uploaded to the Aiforia image processing platform (Aiforia Inc., Cambridge, MA, USA) to quantify mast cells; cytoplasmic granules were highlighted with the TB stain in order to confirm cell identity and neutrophils via deep learning convolutional neural networks (CNNs) and supervised learning (see **Supplementary Material** for validation of AI models) models that were developed and validated by Dr. Lindsey A Smith (Aiforia Technologies), Dr. Syeda Areeha Batool (Aiforia Technologies), and Dr. L. Garry Adams. The number of mast cells and PMNs was calculated by dividing the total mast cell count by the area (mm^2^); both values were provided by the AI.

### 2.12 Effect of r*Ixs*S41 on *B. burgdorferi* colonization of mice

Mice were anesthetized with Ketamine/Xylazine (87.5/12. 5 mg/kg) cocktail via IP. The dorsal thoracic area was shaved with hair clippers and thoroughly cleansed with 70% ethanol. Three mice per treatment were intradermally injected using a 27G, ½″ needle with 1 × 10^7^ *B. burgdorferi* spirochetes strain 31 MSK_5_ (Labandeira-Rey and Skare, 2001; Labandeira-Rey et al., 2003) in BSK-II mixed with or without r*Ixs*S41 (25 µg), C48/80 (1 µg), or mix (r*Ixs*S41 [25 µg] and C48/80 [1 µg]) (100 µL total volume). The relative number of spirochetes and the amount of *Ixs*S41 secreted by ticks are unknown; however, a 2002 study showed that 2-mm skin biopsies from untreated LD patients with erythema migrans (EM) lesions had an average of 3,381 ± 544 spirochetes per specimen (Liveris et al., 2002). Therefore, for this experiment, we decided to use a high number of spirochetes to compensate for the high amount of *Ixs*S41. We assumed that spirochetes injected at high numbers could survive the pro-inflammatory condition that was induced by C48/80.

Following needle inoculation, the tarsal joint swelling was measured using a caliper and ear skin tissue was sampled at 7 days post-inoculation (closer to the duration of tick feeding), followed by euthanasia at 14 days and a second round of tarsal joint measurement. Immediately after, necropsy was performed to harvest brain, heart, tibiotarsal joint, skin, and spleen. All organs were subjected to gDNA extraction using the DNeasy Blood & Tissue kit (Qiagen, Germantown, MD, USA), which was later used as template (60 ng of each organ) for quantitative (q) PCR analysis using published *Bb flaB* primers: F: 5′-TCTTTTCTCTGGTGAGGGAGCT-3′ and R: 5′-TCCTTCCTGTTGAACACCCTCT-3′ and murine β-actin primers: F: 5′-CAAGTCATCACTATTGGCAACGA-3′ and R: 5′-CCAAGAAGGAAGGCTGGAA AA (Van Laar et al., 2016) at 0.3 µM concentration in 50 µL of qPCR reactions using iTaq Universal SYBR Green Supermix (Bio-Rad, Hercules, CA, USA). Cultured *Bb* gDNA (19.6 ng/µL) and mouse gDNA (131.1 ng/µL) were 5-fold diluted and used as standards. The Bio-Rad CFX96 was used with the following conditions: one cycle of 50°C for 2 min and activation at 95°C for 10 min, followed by 40 cycles of denaturation at 95°C for 15 s, and annealing/extension at 60°C for 1 min. Quantification of the *flaB* gene target was done using the delta delta Ct method (2^−ΔΔCt^) as described previously (Livak and Schmittgen, 2001).

### 2.13 Statistical analysis

All statistical analyses were conducted using Prism 9 (GraphPad Software Inc, San Diego, CA, USA). Unpaired ***t***-test was used to determine the statistical significance of the r*Ixs*S41 effect on complement MAC (2.8). Ordinary one-way ANOVA with Dunnett’s multiple comparisons test was used to determine the significance of r*Ixs*S41 on *B. burgdorferi* survival in Serum Complement Sensitivity Assay (2.9), in Balb/C mice paw edema (2.10), and in co-inoculating r*Ixs*S41 and *B. burgdorferi* with and without C48/80 (2.12). To verify the significance among mast cells and neutrophils that was quantified by AI (2.11), the two-way ANOVA with Tukey’s multiple comparisons test was conducted. A *p*-value ≤ 0.05 was considered statistically significant.

## 3 Results

### 3.1 *Ixodes scapularis* serpin 41 is highly conserved in *Ixodes* spp. and is not redundant

*Ixs*S41 (EEC14235.1 or XP_002402368), which we previously described among 45 tick serpins (Mulenga et al., 2009), was found among tick saliva proteins that were abundantly secreted by *I. scapularis* nymphs infected with *B. burgdorferi* (Kim et al., 2021). In GenBank, the EEC14235.1 sequence is truncated at the C-terminal end (22 amino acid residues are missing). Here, we successfully used 3′ RACE to clone the full-length cDNA. Comparative nucleic acid and amino acid sequence analyses reveal that *Ixs*S41 in this study is 98% and 96% identical to EEEC14235.1 and XP_002402368.4 in GenBank, respectively. Notably, the functional domain reactive center loop (RCL) of *Ixs*S41 cloned in this study has one amino acid difference with *I. scapularis* serpin EEC14235.1 and XP_002402368 at position 12 (L-V) and an additional difference with the latter at position 16 (E-K) (not shown). It is very likely that the amino acid differences between *Ixs*S41 (cloned in this study) and sequences in GenBank (EEC14235.1 and XP_002402368.4) are likely due to sequencing errors as we did not find any amplicons with K and V at positions 16 and 12 in the RCL out of 10 amplicons that were sequenced (not shown).

**Figure 1:**
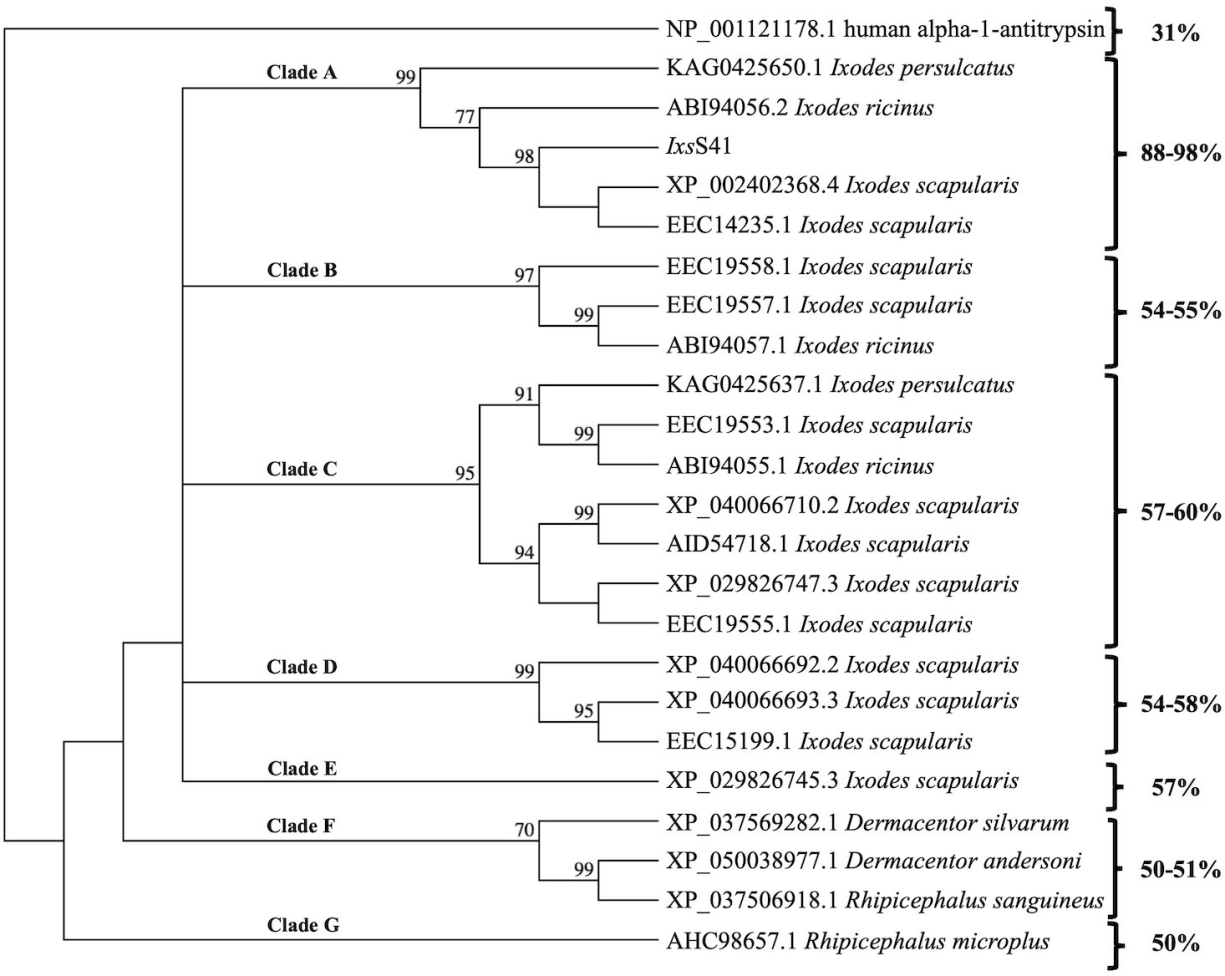
*Ixodes scapularis* (*Ixs*) serpin (S) 41 is conserved in Prostriata but not Metastriata ticks. *Ixs*S41 and other tick serpin amino acid sequences obtained from GenBank were used to construct the phylogeny tree. The neighbor-joining method in MacVector was used with absolute differences detected and bootstrap values set at 1,000 replications. Clades (A–G) represent groups that branch off from the outlier, human alpha-1-antitrypsin.

To gain insight into the sequence relationship of *Ixs*S41 to other tick serpins, we constructed a neighbor-joining phylogeny tree of *Ixs*S41 and 22 other tick serpin sequences that were >50% identical with bootstrap set to 1,000 replications. The phylogeny tree reveals that *Ixs*S41 is not redundant in *I. scapularis* but is conserved in *Ixodes ricinus* and *I. pesulcatus*. Pairwise alignment shows that *Ixs*S41 amino acid sequence is up to 54% identical to other *I. scapularis* serpins (**Figure 1**). However, *Ixs*S41 amino acid sequence is 88% and 94% identical to *I. persulcatus* (KAG0425650.1) and *I. ricinus* (ABI94056.2), respectively, indicating high conservation in other *Ixodes* spp. ticks. Notably, pairwise amino acid sequence alignment shows that the RCL of *Ixs*S41 (EEGTEAAAATGVVIVP**Y**SLGP) has two amino acid differences at positions 13 (E-Y) and 9 (A-T) with *I. ricinus* ABI94056.2 RCL (EEGTVAAATTGVVIVP**Y**SLGP) and one difference at position 13 (E-Y) with KAG0425650.1 RCL (EEGTVAAAATGVVIVPYSLGP) (SF1). Finally, *in silico* analysis predicted two N-linked potential sites at amino acids 88 (NSTL) and 249 (NLTI) and two O-linked potential sites at amino acids 76 and 79 (not shown).

### 3.2 *Pichia pastoris* expressed recombinant (r) *Ixs*S41 is glycosylated

**Figure 2:**
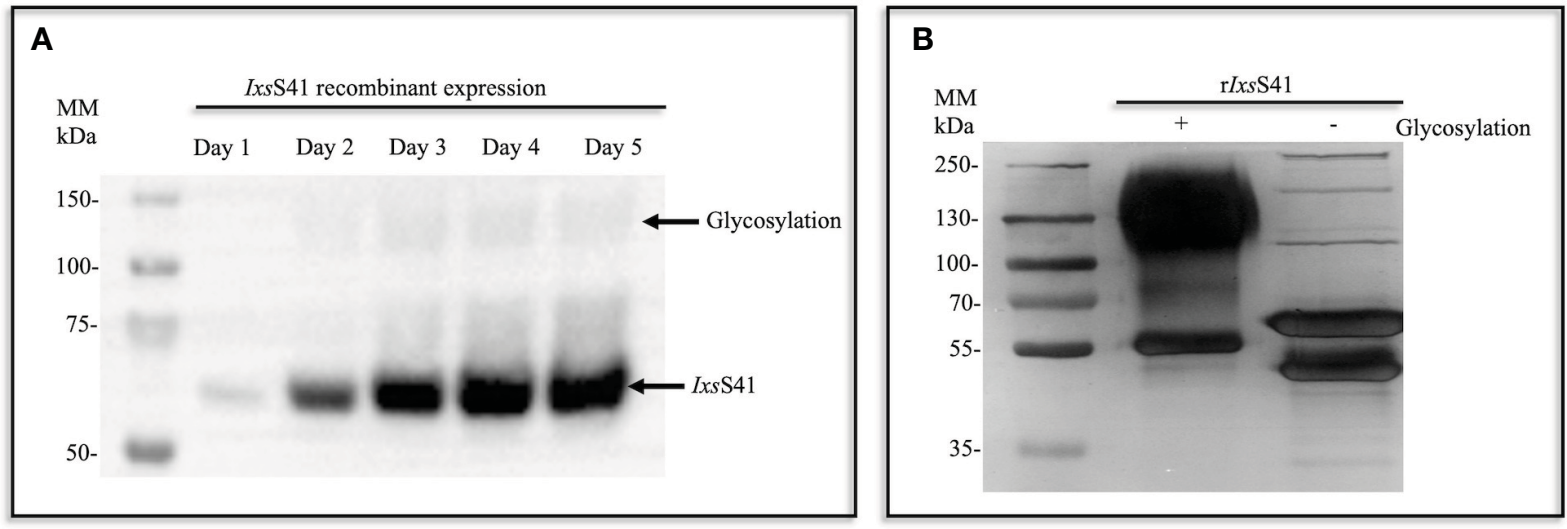
Recombinant (r) *Ixs*S41 expressed as a glycoprotein in *Pichia pastoris*. **(A)** In a small-scale expression (10 mL total), C-terminus histidine-tagged r*Ixs*S41 was expressed in *P. pastoris* X-33 strain for 5 days (methanol induced). Expression was verified by immunoblotting using the mouse antibody to the histidine tag HRP-conjugated antibody. **(B)** Large-scale expression was methanol induced in 1-L batches and metal ion affinity purified under native conditions. Subsequently, affinity-purified r*Ixs*S41 was deglycosylated to remove both N- and O-linked glycans for 1 h at 37°C.

Pilot expression showed that r*Ixs*S41 was methanol induced within 24 h and increased in abundance over 5 days (**Figure 2A**). Subsequently, large-scale expression (1 L batches) was methanol induced for 5 days (**Figure 2B**), and affinity purified using Nickel affinity columns as published (Kim et al., 2015; Tirloni et al., 2019). As shown (**Figure 2B**; lane 1), the protein band of affinity**-**purified r*Ixs*S41 was diffuse with molecular weight ranging between ~55 kDa and slightly under 250 kDa, which is higher than the calculated 42 kDa for *Ixs*S41.

Treatment with deglycosylation enzyme mix to remove both N- and O-linked glycans confirmed that r*Ixs*S41 is expressed as a glycosylated protein in *P. pastoris* as the molecular weight of the deglycosylated (under non-denaturing conditions [**Figure 2B**; lane 2; **Supplementary Figure 1B**]) r*Ixs*S41 was reduced to two major protein bands at ~50–55 kDa and three high-molecular-weight protein bands at 100 kDa, 130 kDa, and 250 kDa. However, when treated under denaturing conditions, we observed two additional predominant bands at 37 and 45 kDa (**Supplementary Figure 1C**). Thus, to gauge the purity of r*Ixs*S41, we used *in gel* LC-MS/MS analysis to identify proteins in each of the bands (**Supplementary Figure 1**). This analysis showed that r*Ixs*S41 was the most predominant among the protein bands that were analyzed as determined by the normalized spectra abundance factor (NSAF) (**Supplementary Table 1**).

### 3.3 Rabbits fed on by *B. burgdorferi*-infected *I. scapularis* nymphs generate an apparently high antibody titer to r*Ixs*S41

**Figure 3:**
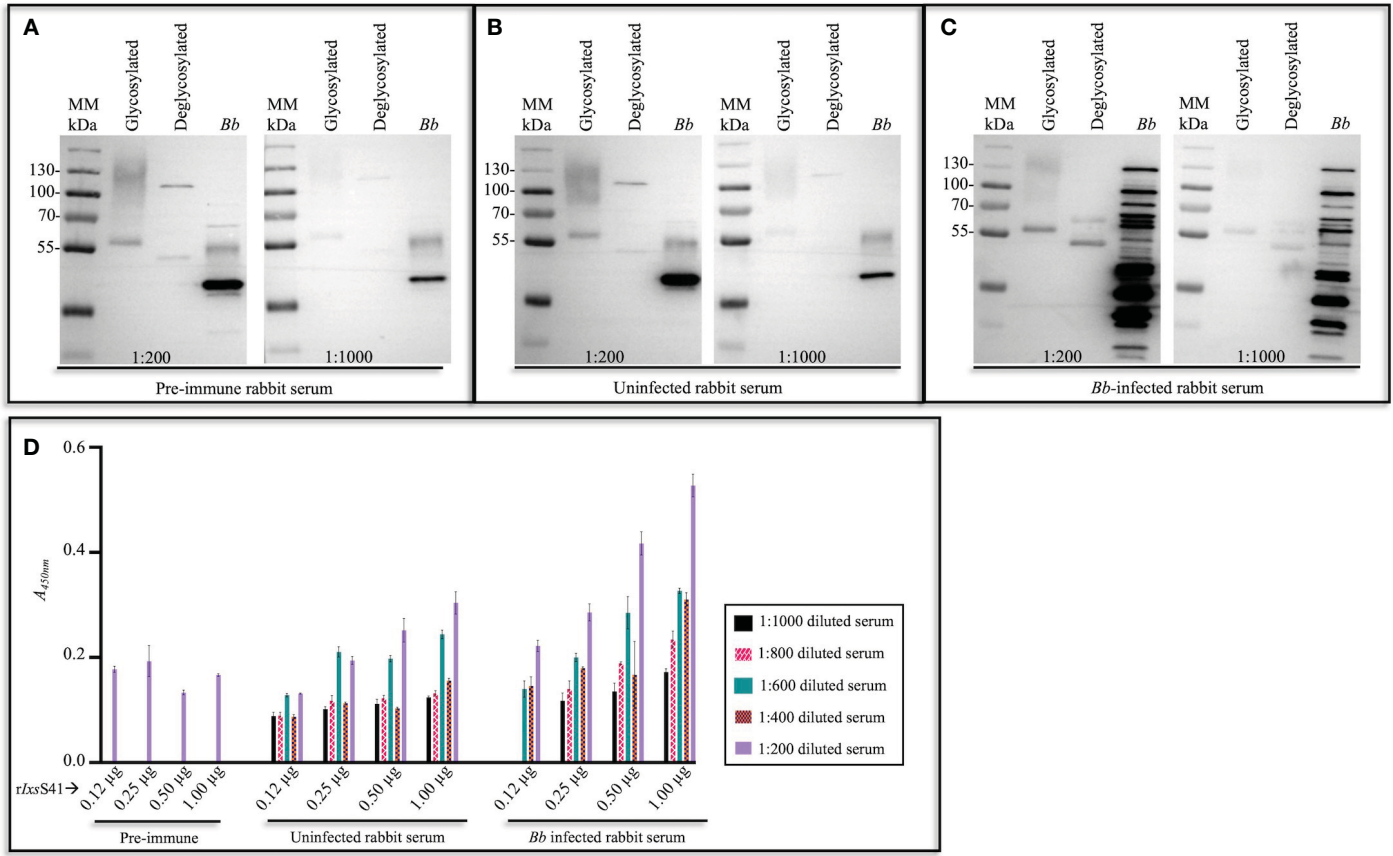
*I scapularis* nymph-secreted *Ixs*S41 elicits antibody response in rabbit. Affinity-purified and empirically optimized r*Ixs*S41 (2 µg) (glycosylated [non-deglycosylated] and deglycosylated) were resolved on a 10% SDS-PAGE gel and subjected to immunoblotting using pre-infestation (or pre-immune) rabbit serum **(A)** and sera of rabbits that were twice infested with uninfected **(B)** and *B. burgdorferi (Bb)*-infected **(C)** *I scapularis* nymphs diluted at 1:200 and 1:1,000. Goat antibody to rabbit IgG (HRP conjugated) was used as secondary antibody (1:2,000). *Bb* protein extract (2 µg) was included as control to distinguish between rabbit antibody to saliva proteins of uninfected and *Bb-*infected *I scapularis* nymphs. **(D)** A 96-well plate was coated with increasing amounts (0.12–1.00 µg) of r*Ixs*S41, washed with PBS-T, and blocked with 5% skim milk in PBS-T. Rabbit serum from pre-infestation (pre-immune) and post-infestation with uninfected or *Bb*-infected *Ixodes scapularis* nymph ticks was used at varying dilutions (1:200–1:1,000) to probe r*Ixs*S41. After washing, HRP-conjugated goat anti-rabbit IgG (1:2,000) was added, plates were washed, and 1-Step Ultra TMB-ELISA substrate was added. Signal was detected with a plate reader and the data are presented as mean A_450nm_ ± SEM for each r*Ixs*S41 amount and sera dilution.

Since *Ixs*S41 was identified among tick saliva proteins that are highly secreted by *Bb-*infected nymphs (Kim et al., 2021), we sought to determine its immunogenicity. We found that *Ixs*S41 is among tick saliva proteins that elicit a rabbit immune response to saliva proteins of *Bb-*infected nymphs (**Figure 3**). Western blot analysis shows that binding intensities to r*Ixs*S41 of immune sera of rabbits that were fed on by uninfected *I.* scapularis nymphs (**Figure 3B**) were weaker than immune sera of rabbits that were fed on by *B. burgdorferi-*infected ticks (**Figure 3C**). These findings were reproduced in our ELISA data (**Figure 3D**). As shown, *A*_450nm_ (optical density) levels for uninfected sera ELISA were lower than infected sera ELISA confirming that rabbits that were fed on by *Bb-*infected ticks had high antibody titer to r*Ixs*S41. We would like to note that sera of rabbits before they were fed on by ticks (or pre-immune sera) was non-specifically binding to r*Ixs*S41. However, non-specific binding was significantly diminished when the antibody was diluted 1,000-fold. It is also notable that at high primary antibody concentration (1:200 dilution), binding to deglycosylated r*Ixs*S41 (blue arrows ↑) is much weaker (faint) when probing with pre-immune (**Figure 3A**) or uninfected rabbit serum (**Figure 3B**), compared to the strong (darker) band observed with *Bb*-infected rabbit serum (**Figure 3C**). Similarly, when the same sera type is used at a more diluted concentration (1:1,000) to probe deglycosylated r*Ixs*S41 (red arrows ↑), binding to the r*Ixs*S41 protein backbone is only observed when using *Bb*-infected rabbit serum. Considering that binding to the glycosylated version of r*Ixs*S41 is observed across all three sera types, we suspect that glycans contributed to the nonspecific binding.

It is also important to note to that pre-immune and uninfected rabbit serum also non-specifically bound to some *Bb* proteins (marked by asterisks [*]): multiple other bands are observed when using *Bb*-infected rabbit serum.

### 3.4 r*Ixs*S41 inhibits pro-inflammatory proteases

**Figure 4:**
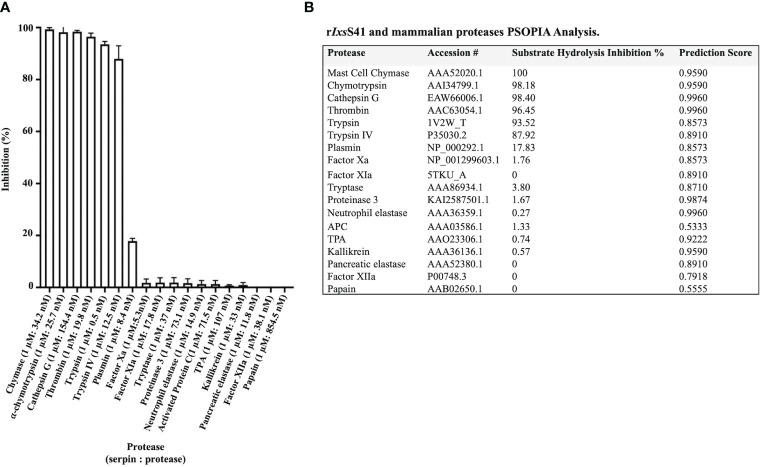
r*Ixs*S41 is an inhibitor of inflammatory and complement system effector serine proteases. **(A)** Excess r*Ixs*S41 (1 µM) was pre-incubated with 18 mammalian proteases for 15 min at 37°C. Subsequently, 0.20 mM final concentration of appropriate peptide chromogenic pNA substrates were added, and hydrolysis was immediately monitored every 11 s at *A*_405nm_ for 15 min using a plate reader. Data are presented as mean percent inhibition of 1 µM of r*Ixs*S41 to protease ± SEM. **(B)** Prediction scores for the interaction between r*Ixs*S41 and all proteases used in substrate hydrolysis assays were obtained using the PSOPIA prediction server (probabilities 0–1.0). Column titled “r*Ixs*S41 Inhibition Percent” is presented in **(A)**.

Substrate hydrolysis assays of 18 mammalian serine proteases confirmed that r*Ixs*S41 (1 µM) inhibited the enzymatic activity of 6 of the 18 tested proteases by nearly 100% (**Figure 4A**). These include chymase (34.2 nM), chymotrypsin (25.7 nM), cathepsin G (154. 4 nM), thrombin (19.8 nM), pancreatic trypsin (0.5 nM), and trypsin IV (12.5 nM). PSOPIA analysis revealed high interaction prediction scores between r*Ixs*S41 and the aforementioned proteases: chymase (0.9590), chymotrypsin (0.9590), cathepsin G (0.9960), thrombin (0.9960), pancreatic trypsin (0.8573), and trypsin IV (0.8910) (**Figure 4B**). Interestingly, PSOPIA analysis revealed moderate to high interaction prediction scores for proteases that were not inhibited by r*Ixs*S41 in our substrate hydrolysis assay (**Figure 4B** [table insert]). PSOPIA analysis shows that r*Ixs*S41 likely interact with neutrophil elastase (0.9960), proteinase 3 (0.9874), kallikrein (0.9590), tissue plasminogen activator ([TPA] 0.9222), factor XIa and pancreatic elastase (0.8910), tryptase (0.8710), plasmin and factor Xa (0.8573), and factor XIIa (0.7918). Consistently, low PSOPIA prediction scores were observed for activated protein C ([APC] 0.5333) and papain (0.5555), proteases that were not inhibited by r*Ixs*S41 like factor H, our non-protease control (**Figure 4B**).

**Figure 5:**
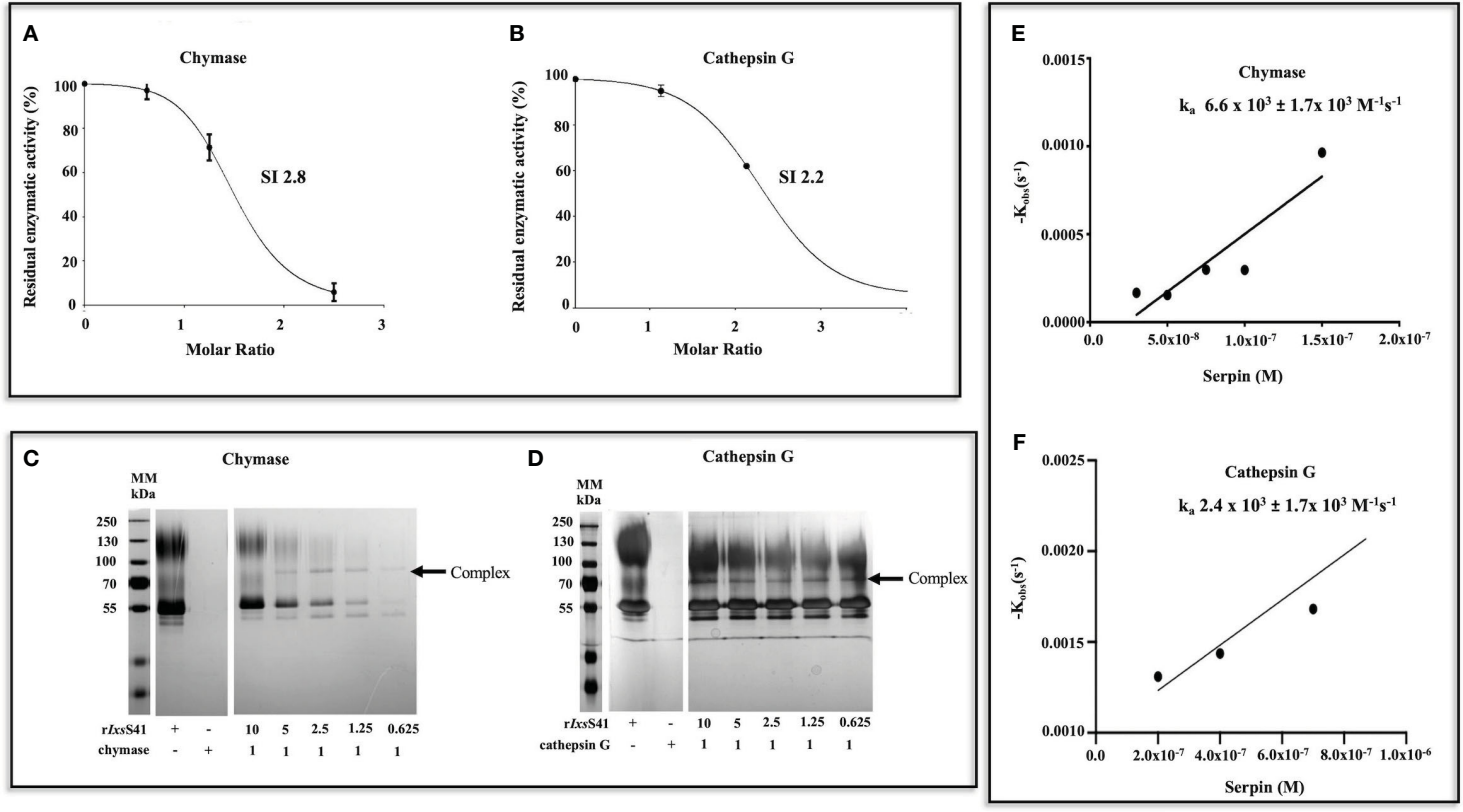
Few molecules of r*Ixs*S41 are needed to inhibit chymase and cathepsin G at a slower rate than is observed with native inhibitors. **(A, B)** SI was estimated by incubating different molar ratios (0–10) of r*Ixs*S41-to-chymase or cathepsin G for 1 h at 37°C. Immediately after, the protease activity was verified with colorimetric substrates and data were plotted as residual enzymatic activity. The SI value was obtained by linear regression of the inhibitory curve in PRISM. **(C, D)** To confirm if r*Ixs*S41 retains typical inhibitory serpin properties of forming SDS and heat-resistant complexes with target proteases, various molar ratios of r*Ixs*S41 (10–0.625)-to-chymase or cathepsin G were pre-incubated for 1 h at 37°C. Thereafter, the reaction was halted with SDS-PAGE reducing sample buffer and heat denatured (95°C for 5 min). Samples were resolved on a 10% SDS-PAGE gel and visualized by silver staining. Arrowheads (←) denote the complexes between r*Ixs*S41 and proteases (chymase and cathepsin G). **(E, F)** The second-order rate constant (*k*_a_) for the inhibition of chymase and cathepsin G **(B)** by r*Ixs*S41 was determined using the discontinuous method. Different amounts of r*Ixs*S41 (25–300 nM against chymase [0.1 µg] or 100–1600 against cathepsin G [0.1 µg]) were pre-incubated for 0–15 min at 37°C with constant amounts of chymase or cathepsin G to obtain the residual enzymatic activity. The rate of chymase or cathepsin G activity was calculated by linear regression analysis. The best fit line of *k_obs_* (pseudo-first-order constant) values were plotted against different amounts of r*Ixs*S41, thus producing the *k*_a_ for chymase and cathepsin G inhibition.

Since molar excess of r*Ixs*S41 was used in substrate hydrolysis, there was the possibility that some of our observations were not physiologically relevant. To resolve this, we next used the stoichiometry of inhibition (SI) analysis to estimate the lowest amount of r*Ixs*S41 required to inhibit one molecule of the target protease by 100%. This analysis estimated that approximately 2.8 and 2.2 molecules of r*Ixs*S41 are needed to 100% inhibit one molecule of chymase and cathepsin G, respectively (**Figures 5A, B**). We observed SI of above 7 against chymotrypsin, thrombin, pancreatic Trypsin, and Trypsin IV (not shown).

A typical inhibitory serpin will form a heat- and SDS-stable complex with its target protease (Marijanovic et al., 2019). Consistently, r*Ixs*S41 formed an irreversible heat- and SDS-stable complex with both chymase and cathepsin G (**Figures 5C, D**). We next used the discontinuous method (Horvath et al., 2011) to estimate the rate of r*Ixs*S41 inhibition (*k*_a_) against both chymase and cathepsin G. This analysis estimated that r*Ixs*S41 inhibited chymase and cathepsin G at a *k*_a_ of 6.6 × 10^3^ ±1.7 × 10^3^ M^−1^ s^−1^ and a *k*_a_ of 2.4 × 10^3^ ±1.7 × 10^3^ M^−1^ s^−1^, respectively (**Figures 5E, F**).

### 3.5 r*Ixs*S41 affects membrane attack complex deposition via the alternative and lectin pathway and blocks complement killing of *B. burgdorferi*

**Figure 6:**
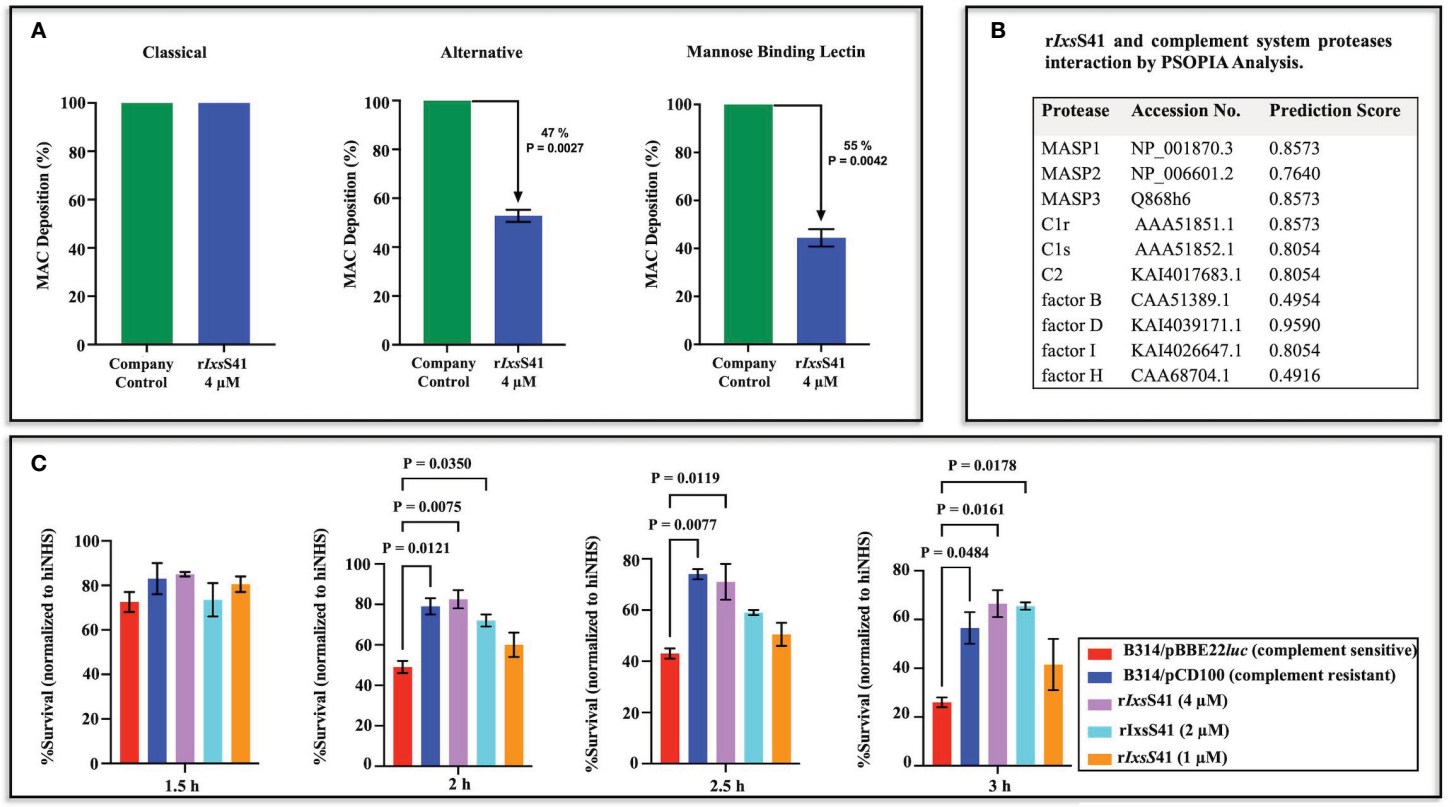
r*Ixs*S41 reduces deposition of membrane attack complex of complement via the alternative and mannose binding lectin pathways and prevents serum complement killing of complement-sensitive *Bb* strain B314/pBBE22*luc*. **(A)** The effect of r*Ixs*S41 on deposition of the membrane attack complex (MAC) via the Classical, Alternative, and Mannose Binding Lectin (MBL) activation pathways of complement was assayed using the WiesLab Complement System kit. Affinity-purified r*IxsS*41 (4 µM) was pre-incubated with human serum for 30 min at 37°C before activating the complement system. Negative (NC) and positive (PC) control sera provided in the kit were incubated with Tris-HCl buffer only. The *A*_405nm_ was obtained after the addition of conjugate and substrate and data were plotted as mean percent of MAC deposition ± SEM of duplicates. Arrowheads (↓) indicate inhibition percent of MAC deposition that was calculated using the formula: 100 − (Sample-NC)/(PC-NC) × 100%. **(B)** Prediction scores for the interaction between r*Ixs*S41 and complement-involved proteases were obtained using the PSOPIA prediction server (probabilities 0–1.0). **(C)** To further confirm r*Ixs*S41’s effect in complement, various amounts of r*Ixs*41 (1-4 µM) or PBS were pre-incubated with normal human serum or heat-inactivated human serum (hiNHS) for 30 min at 37°C. Serum complement-sensitive (B314/pBBE22*luc*) or -resistant (B314/pCD100) *Bb* strains at a concentration of 1 × 10^6^ spirochetes were added and incubated at 32°C. *Bb* survival was scored by counting spirochetes in 10 fields of view under dark field microscopy (40×) at 1.5, 2.0, 2.5, and 3.0 h post-treatment. Data are presented as mean % survival normalized to hiNHS ± SEM.

Although r*Ixs*S41 inhibited blood clotting factor IIa (thrombin), it had no effect against blood clotting (not shown). In the complement assay, r*Ixs*S41 reduced deposition of the MAC via the Alternative (47% reduction *p* = 0.0027) and MBL (55%, *p* = 0.0042) pathways but not the Classical pathway (**Figure 6A**). Consistently, PSOPIA prediction analysis revealed high interaction scores for *Ixs*S41 against proteases in MBL and Alternative complement pathways: MASP1 (0.8573), MASP2 (0.7640), MASP3 (0.8573), C1r (0.8573), C1s (0.8054), C2 (0.8054), factor D (0.9590), and factor I (0.8054) (**Figure 6B**).

Since the MBL pathway of complement is important to *B. burgdorferi* clearance (Sajanti et al., 2015), we next evaluated if r*Ixs*S41 was protective against killing of serum complement-sensitive *B. burgdorferi* strain (B314/pBBE22*luc* [*luc*]) by complement (**Figure 6C**). Consistent with reduced deposition of the MAC, r*Ixs*S41 dose-dependently protected the complement-sensitive *B. burgdorferi* strain from killing by complement. Albeit not statistically significant at the 1.5-h time point, the survival rate of the serum sensitive *luc* strain treated with r*Ixs*S41 was higher than that of the complement-sensitive strain. Likewise, comparable to the resistant strain, the survival rate of the complement-sensitive *luc* strain incubated with 4 and 2 µM of r*Ixs*S41 was significantly higher than the *luc* strain incubated with PBS at *p* = 0.0207, *p* = 0.0158, or *p* = 0.0011 at the 2.0-, 2.5-, and 3.0-h time points, respectively. The survival rate of serum-sensitive *luc* strain treated with 1 µM of r*Ixs*S41 was apparently higher than the sensitive strain but not statistically significant at all time points.

### 3.6 r*Ixs*S41 inhibits compound 48/80-induced paw edema in BALB/c mice

**Figure 7:**
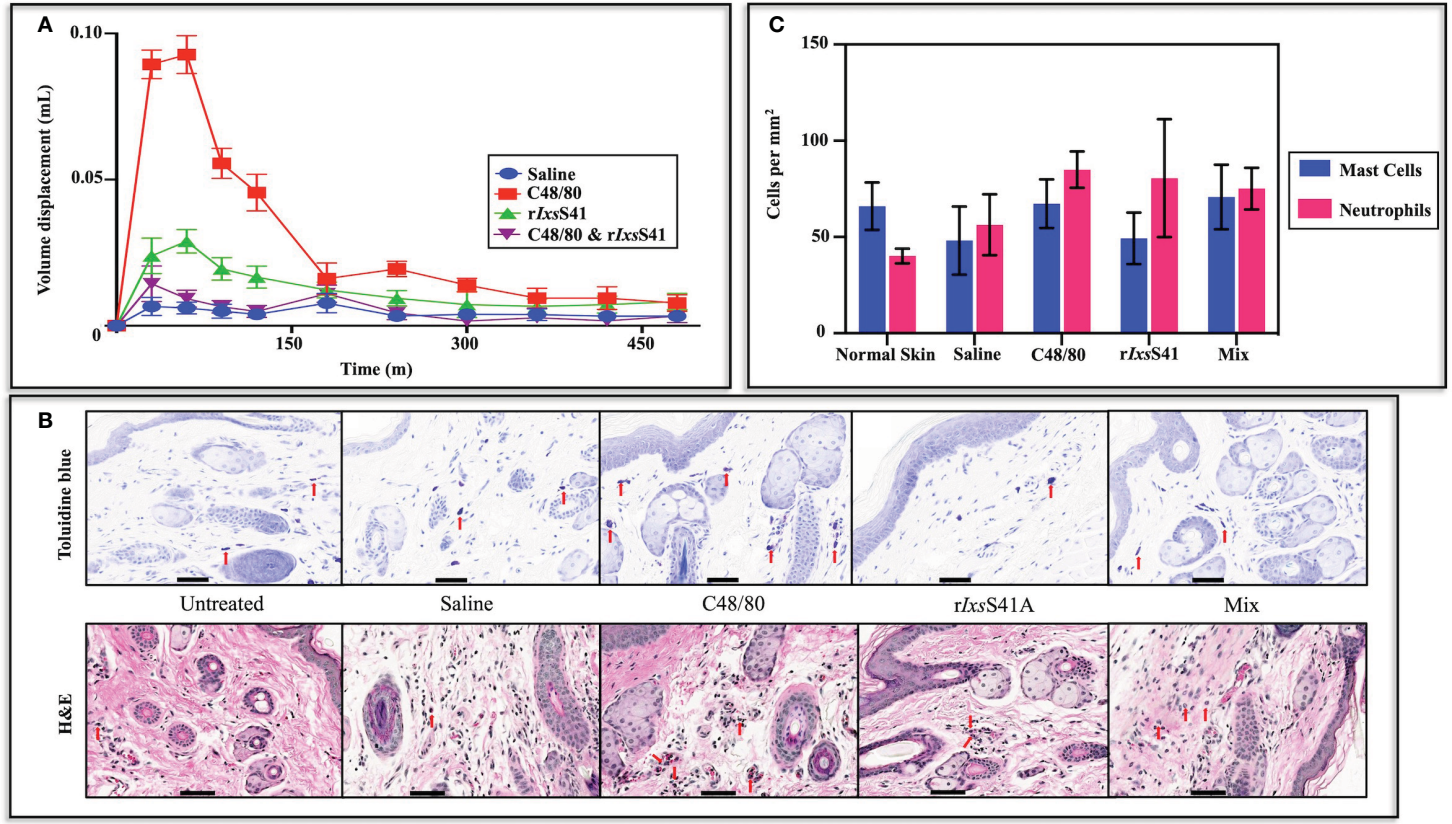
r*Ixs*S41 inhibited chymase-induced BALB/c mice paw edema but did not significantly alter the mast cell profile or neutrophil influx into the injection site with or without an inflammation agonist. **(A)** Twelve retired female BALB/c mice, divided into four groups, were intradermally injected (25 µL volume) on the left basal footpad with saline, compound 48/80 (1 µg of mast cell degranulator), r*Ixs*S41 (25 µg), or mixture (compound 48/80; 1 µg and r*Ixs*S41; 25 µg) in 20 µL total volume. Mouse paw swelling (inflammation) was measured using a digital plethysmometer every 30 min for the first 120 min (2 h) and every 60 min thereafter through 8 h. The data are presented as mean volume displacement (mm) readings for 480 min (8 h) ± SEM. **(B)** Skin biopsies of basal footpad of BALB/c mice (three per treatment) were taken with a scalpel at 50 min after injection (25 µL volume) with saline, compound 48/80 (1 µg), r*Ixs*S41 (25 µg), or Mix (compound 48/80, 1 µg and r*Ixs*S41, 25 µg). Tissues were fixed in 15 mL of 4% paraformaldehyde and paraffin embedded. Subsequently, slides were stained with toluidine blue for mast cell and hematoxylin & eosin (H&E) for polymorphonuclear leukocyte (PMN) identification. Red arrowheads (↓) indicate mast cells and PMNs. **(C)** Stained slides were uploaded to Aiforia image processing and management platform for mast cell and neutrophil quantification by convolutional neural networks (CNNs) and supervised learning. Data are plotted as mean mast cell or neutrophil count per mm^2^ ± SEM. Scale bar on images **(A, B)** = 20 µm.

Prompted by findings that r*Ixs*S41 inhibited chymase (**Figures 4** and **5**), we investigated its effect on chymase-mediated paw edema that was induced by mast cell degranulator, C48/80 (Irman-Florjanc and Erjavec, 1983; Chatterjea et al., 2012). The paw edema assay in BALB/c mice validated that r*Ixs*S41 is a functional anti-inflammatory protein *in vivo* using the paw edema assay in BALB/c mice (**Figure 7A**). In the absence of r*Ixs*S41, C48/80 caused swelling of treated hind paws within 30–50 min after injection. However, co-injection of C48/80 (1 µg) with r*Ixs*S41 (25 µg) significantly reduced swelling of treated paws by 85% (*p* = 0.0037). Peak volume displacement reading reduced from 0.07 (0.07± 0.0068) in C48/80 to 0.01 (0.01 ± 0.0033) in r*Ixs*S41 + C48/80 injected paws at *p* = 0.0037 (**Figure 7A**). The results presented are an average of six mice per treatment.

We next sought to understand if the anti-inflammatory effect of r*Ixs*S41 affected the profiles of pro-inflammatory cells in the inflamed site using Toluidine blue and H&E staining (**Figure 7B**). Quantification of mast cells and neutrophils with deep learning convolutional neural networks (CNNs) and supervised learning (Aubreville et al., 2020; Allier et al., 2022) revealed no significant differences in the number of mast cells or neutrophils per mm^2^ in injected sites with any of the treatments. However, similar to saline (48.13 ± 17.73), mast cells per mm^2^ in r*Ixs*S41-injected sites (49.33 ± 13.38) were apparently lower than in normal skin (untreated control [65.96 ± 12.30]) and C48/80-injected (67.36 ± 12.62) and r*Ixs*S41- and C48/80-injected sites (Mix [70.86 ± 16.75]). In case of neutrophils, untreated skin had apparently fewer cells per mm^2^ (40.14 ± 3.834) than saline (56.33 ± 15.83), C48/80 (84.97 ± 9.407), r*Ixs*S41 (80.61 ± 30.63), and Mix (75.16 ± 10.79) (**Figure 7C**).

### 3.7 r*Ixs*S41 differentially affects *B. burgdorferi* colonization of C3H/HeN mice

**Figure 8:**
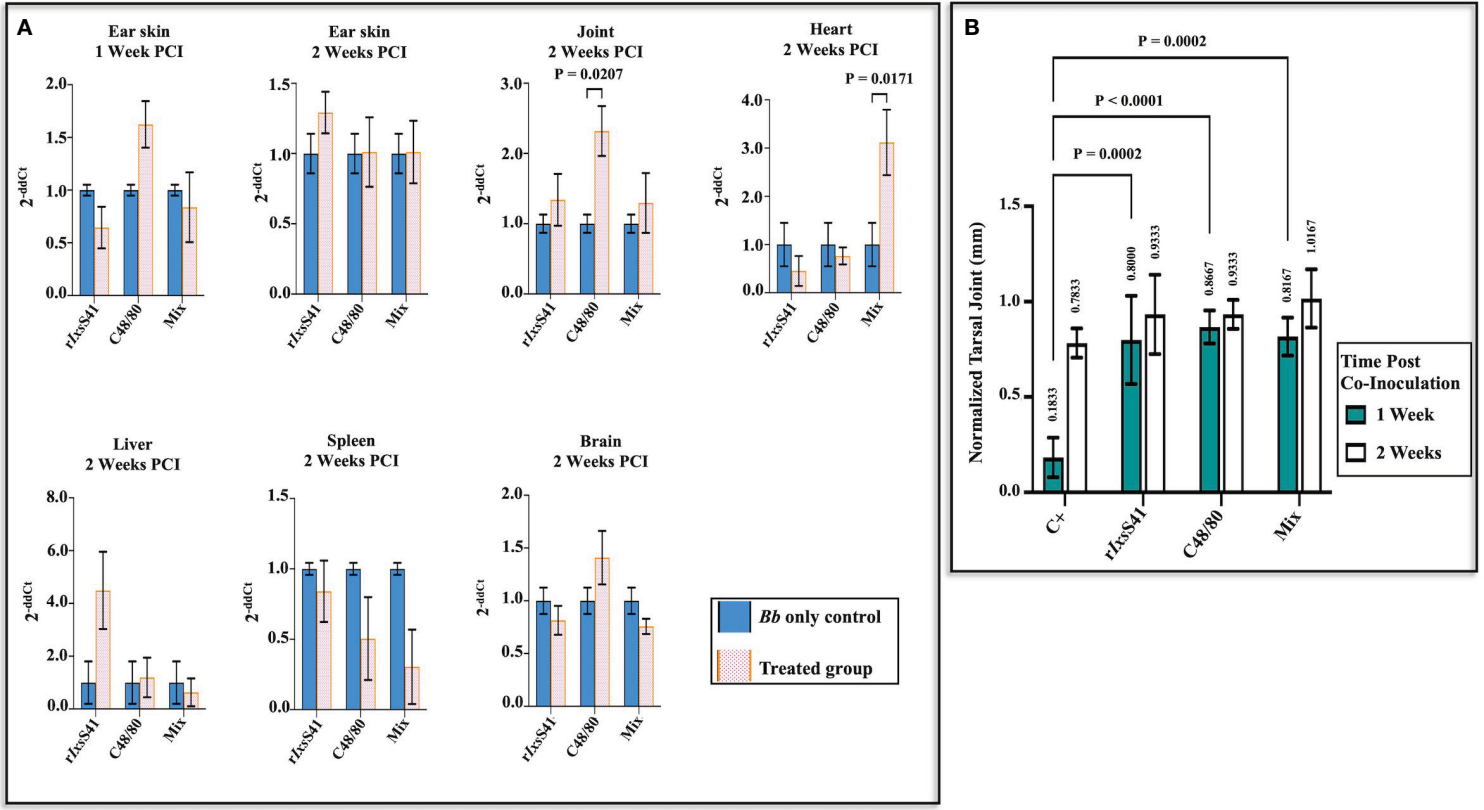
Co-inoculating C3H/HeN mice with r*Ixs*S41 and *B burgdorferi* enhances spirochete colonization of the heart while C48/80 promotes joint colonization, with increased tarsal joint swelling on mice treated with r*Ixs*S41 and C48/80 at 1 week post-infection. Retired female C3H/HeN mice (three per treatment) were intradermally inoculated with 1 × 10^7^ *B burgdorferi Bb* with or without r*Ixs*S41 (25 µg), C48/80 (1 µg), or Mix (compound 48/80 [1 µg] and r*Ixs*S41 [25 µg]) on the dorsal scapular area. **(A)** A week following inoculation, ear skin tissue was sampled, and 2 weeks later, mice were euthanized and ear skin, joint, heart, liver, spleen, and brain organs were collected. The gDNA of collected tissues was extracted and used for qRT-PCR quantification of *Bb* using *Bb flab* and murine *β*-actin (internal reference gene) primers. The data are presented as fold change (2^−ΔΔCt^) of *Bb flaB* gene among treatments compared to the *Bb*-only group. **(B)** The swelling of both tarsal joints was measured at 1 and 2 weeks after infection. The data are presented as the mean of tarsal swelling normalized against tarsal joint measurement of negative control mice (media inoculated) ± SEM.

Given the anti-inflammatory effects of r*Ixs*S41 (**Figure 7A**), we investigated the effects of r*Ixs*S41 on *B. burgdorferi* colonization of C3H/HeN mice. **Figure 8** summarizes the effect of r*Ixs*S41 and C48/80 on *B. burgdorferi* colonization of C3H/HeN organs. Compared to *B. burgdorferi-*only control, C48/80 (1 µg) enhanced *B. burgdorferi* colonization of ear skin at 1 week post-intradermal inoculation by 0.62 ± 0.22-fold and 1.32 ± 0.40-fold in joints (*p* = 0.02), and 0.40 ± 0.25-fold in brain at 2 weeks post-inoculation. Likewise, C3H/HeN that intradermally received r*Ixs*S41 (25 µg) and *B. burgdorferi* had a high spirochete load by 3.50 ± 1.50-fold in liver while mice receiving the mixture r*Ixs*S41(25 µg) and C48/80 (1 µg) had significantly higher spirochete load (2.12 ± 0.68-fold) in heart tissue (*p* = 0.01). No apparent or significant increase in *B. burgdorferi* colonization was seen in skin and spleen 2 weeks post**-**inoculation. It is also notable that the spirochete load was reduced by 0.50 ± 0.29-fold and 0.70 ± 0.26-fold in spleens of mice that received C48/80 and the mixture of r*Ixs*S41 and C48/80, respectively.

**Figure 8B** summarizes tarsal joint swelling at 1 and 2 weeks after intradermally co-injecting mice with 1 × 10^7^ spirochetes mixed with or without r*Ixs*S41 (25 µg), C48/80 (1 µg), or r*Ixs*S41 and C48/80 mixture. As shown, at 1 week post-inoculation, tarsal swelling of control mice that had received spirochetes only was significantly lower than all other treatment groups. However, at 2 weeks post-infection, differences in tarsal swelling were modestly apparent but not statistically significant.

## 4 Discussion

Most published studies characterized tick serpins that are secreted by uninfected ticks (Sugino et al., 2003; Mulenga et al., 2007; Prevot et al., 2007; Yu et al., 2013; Bakshi et al., 2018; Xu et al., 2020). Here, we demonstrate that *Ixs*S41, which is abundantly secreted by *I. scapularis* nymphs infected with *B. burgdorferi* (Kim et al., 2021), is not redundant and conserved among *Ixodes* spp. ticks, but not in Metastriata ticks, indicating that this protein is important in *Ixodes* spp. tick physiology. Most tick serpins are part of clusters of highly identical serpins that may represent redundant molecular systems (Mulenga et al., 2009; Porter et al., 2015), and thus could diminish their utility as target antigens in anti-tick vaccines. Therefore, our comparative sequence analysis finding that *Ixs*S41 did not show amino acid identity levels of above 60% to other *I. scapularis* serpins indicates that this serpin is not redundant and represents a promising candidate for a tick-antigen-based vaccine to prevent *B. burgdorferi* transmission. This view is supported by our Western blot and ELISA analyses that confirmed *Ixs*S41 was immunogenic as indicated by the reaction of antibodies to saliva proteins of both uninfected and *B. burgdorferi-*infected *I. scapularis* nymphs reacted with r*Ixs*S41. Since *Ixs*S41 is abundantly secreted by *I. scapularis* nymphs infected with *B. burgdorferi* (Kim et al., 2021), the apparent high anti-r*Ixs*S41 antibody levels produced by rabbits that were infested by infected ticks were not surprising. Our finding that sera of rabbits that were infested by uninfected ticks had stronger binding to glycosylated r*Ixs*S41 compared to deglycosylated r*Ixs*S41 is intriguing. It would be interesting to investigate if uninfected and *B. burgdorferi*-infected ticks secrete native *Ixs*S41 with different glycosylation levels.

Mature r*Ixs*S41 protein is 94% identical to its homolog in *I. ricinus*, IRIS-2 (ABI94056.2) (Chmelar et al., 2011), with 2 of the 12 amino acid differences located in the functional reaction center loop (RCL) domain at positions 13 (E-Y) and 9 (A-T). Intriguingly, the inhibitory profiles of r*Ixs*S41 and IRIS-2 were similar despite the two amino acid differences in the RCL with different physical properties. Glutamate (E) and alanine (A) residues in IRIS-2 RCL are hydrophilic while tyrosine (Y) and threonine (T) residues in *Ixs*S41 are hydrophobic (PierceProteinMethods). Despite these differences, both r*Ixs*S41 and IRIS-2 (Chmelar et al., 2011) were inhibitors of chymase, cathepsin G, chymotrypsin, pancreatic trypsin (including trypsin IV for r*Ixs*S41), and thrombin. Our findings are also notable because the two recombinant proteins were expressed in yeast (r*Ixs*S41) where posttranslational modification of recombinant proteins occur and is absent in bacteria (IRIS-2) (Sahdev et al., 2008; Mattanovich et al., 2012; Rosano and Ceccarelli, 2014). It would be interesting to investigate the functional significance of posttranslational modification in functions of tick saliva serpins.

Although not empirically validated, we suspect that native *Ixs*S41 will be secreted in low quantities during tick feeding. Therefore, the finding that r*Ixs*S41 inhibited chymase and cathepsin G at respectively low r*Ixs*S41-to-protease molar ratios (or stoichiometry inhibition) of 2.2 and 2.8 strongly suggests that our data approximate physiological events at the tick feeding site. Under physiological conditions, a typical serpin inhibits the activity of its cognate protease with SI closer to 1, rate of reaction (*k*_a_) ≥ 10^5^ M^−1^ s^−1^, and forms heat-stable complexes with cognate target protease (Gettins, 2002; Horvath et al., 2011). In this study, *k*_a_ of 2.4 × 10^3^ M^−1^ s^−1^ (chymase) and 1.7 × 10^3^ M^−1^ s^−1^ (cathepsin G) were slower than what is observed under native conditions (Gettins, 2002; Horvath et al., 2011). This slower reaction rate could be explained by assuming that the reaction conditions in this study only approximate those that occur at the tick bite site. Notably, some of the mammalian serpins such as antithrombin, heparin cofactor II, and protein C inhibitor need to bind glycosaminoglycans (GAGs) to increase potency and rate of reaction, and thus achieve maximal protease inhibition (Li et al., 2004; Tollefsen, 2010; Rein et al., 2011; Lindahl et al., 2015; Sobczak et al., 2018). On this basis, it is possible that r*Ixs*S41 requires binding to a yet unknown cofactor to optimize its rate of reaction. The need for a cofactor may also explain the high SI (more than 7) for r*Ixs*S41 inhibition of chymotrypsin, pancreatic trypsin, blood clotting factor II (thrombin), and trypsin IV. Likewise, the need for a cofactor could explain the fact that proteases that were not inhibited by r*Ixs*S41 in substrate hydrolysis were predicted to significantly interact with *Ixs*S41 on PSOPIA. Collectively, these findings warrant further investigations.

Chymase and cathepsin G are released by mast cells and neutrophils (Pham, 2008; Dai and Korthuis, 2011; Glatz et al., 2017; Zamolodchikova et al., 2020), the two pro-inflammatory immune cells that are active in the tick feeding site early on (Heinze et al., 2012; Hermance and Thangamani, 2018). Both cathepsin G and chymase play important roles in amplifying the inflammatory response through activation of signaling receptors such as cleaving protease-activated receptors (Steinhoff et al., 2005; Grant et al., 2007) and activation or production of chemokines and cytokines (Meyer-Hoffert, 2009; Mantovani et al., 2011; Varvara et al., 2018), which, in turn, attract other immune cells to the site of inflammation such as the tick feeding site (Heinze et al., 2012; Blisnick et al., 2017). When mast cells are activated by C48/80, their content, including chymase, is released to the extracellular space, leading to an inflammatory response (Dai and Korthuis, 2011; Galli et al., 2020). Our finding that r*Ixs*S41 blocked C48/80-induced paw edema demonstrates that r*Ixs*S41 is a functional inhibitor of chymase *in vivo.* It is also notable that the major pro-inflammatory protease that is released by degranulating mast cell is tryptase (Thomas et al., 1998; Algermissen et al., 1999; Payne and Kam, 2004; Caughey, 2007), which, interestingly, was not inhibited by r*Ixs*S41.

Neutrophils respond to injury and infection by infiltrating the inflamed site from blood circulation to drive the inflammatory process and clear the invading pathogens (Mantovani et al., 2011). Albeit not statically significant, our histopathology data indicate that injecting C48/80 and r*Ixs*S41 may have modestly increased neutrophil influx into the inoculation site. This is in contrast to IRS-2, which inhibited neutrophil influx into the inflamed site as revealed by increased myeloperoxidase activity (Chmelar et al., 2011), which is released by invading neutrophils to clear invading microbes (Arnhold, 2020).

Since native *Ixs*S41 was secreted at high abundance by nymphs infected with *B. burgdorferi* (Kim et al., 2021), we examined the roles of this protein in *B. burgdorferi* colonization of the host. Consistent with reduced deposition of the MAC via the Alternative and MBL pathways of complement, r*Ixs*S41 protected serum complement-sensitive *B. burgdorferi* strain (B314/pBBE22*luc*) from being killed by complement in a dose-dependent manner. Of note, MBL is known to play an important role in *B. burgdorferi* clearance (Sajanti et al., 2015; Coumou et al., 2019). Based on our data, the mechanism underlying r*Ixs*S41 protection of *B. burgdorferi* from complement killing is unknown. However, it is possible that *Ixs*S41 inhibits complement cascade pathways via inhibition of complement system proteases that apparently interacted with r*Ixs*S41 as revealed by PSOPIA analysis. These data warrant further investigations. Both chymase and cathepsin G that are strongly inhibited by r*Ixs*S41 are involved in auxiliary activation of the complement system (Huber-Lang et al., 2018). Both chymase and cathepsin G activate (cleave) C3, leading to C3a and C3b, with the latter forming C5 convertase cleaving C5 into C5a (a chemoattractant) and C5b (which initiates assembly of the MAC) (Rawal and Pangburn, 1998; Huber-Lang et al., 2018). It is possible that r*Ixs*S41 inhibition of cathepsin G and chymase indirectly affected the quality of the deposited MAC, leading to survival of *B. burgdorferi.* Likewise, trypsin, chymotrypsin, and thrombin, which are also inhibited by r*Ixs*S41, albeit with low efficiency, contribute to auxiliary activation of complement (Huber-Lang et al., 2018).

Traditionally, RNAi silencing has been harnessed for understanding the significance of tick proteins in feeding and transmission of pathogens (Hajdusek et al., 2009; Bakshi et al., 2018; Kim et al., 2020a; Oleaga et al., 2022). Here, we used a different approach, as we co-inoculated *B. burgdorferi* with r*Ixs*S41 in the presence or absence of the inflammation agonist, C48/80 (Paton, 1951; Irman-Florjanc and Erjavec, 1983; Chatterjea et al., 2012). Our findings indicate that *Ixs*S41 likely promoted dissemination of *B. burgdorferi* from its site of inoculation into the liver while r*Ixs*S41 suppression of C48/80-induced inflammation markedly promoted *B. burgdorferi* colonization of the heart, but not other organs. The finding that co-inoculation of *B. burgdorferi* with C48/80, an inflammation agonist, significantly promoted *B. burgdorferi* colonization of the skin and joint (also seen as joint swelling) was surprising. Our expectation was for the inflammation triggered by C48/80 to significantly suppress *B. burgdorferi* colonization of the organs. However, *B. burgdorferi* has been found to express pro-inflammatory cytokines and other markers when incubated with immune cells (Straubinger et al., 1998; Giambartolomei et al., 1999), injected into the skin (Müllegger et al., 2000; Petnicki-Ocwieja and Kern, 2014), or synovial fluid (Gross et al., 1998; Yang et al., 2008), suggesting that the spirochete flourishes in pro-inflammatory environments. If *B. burgdorferi* flourishes during inflammation, the roles of anti-inflammatory tick saliva proteins such as *Ixs*S41 in *B. burgdorferi* transmission will require further investigation. It is also possible that the amounts of r*Ixs*S41 used in this study did not approximate levels that are secreted by ticks, and thus our reaction conditions may not have been optimal.

In summary, this study revealed that the serpin (*Ixs*S41) that is highly secreted by *B. burgdorferi*-infected *I. scapularis* nymphs is an anti-inflammatory protein that protected *B. burgdorferi* from complement-mediated killing. Our findings warrant further investigations on the role(s) of *IxsS*41 in *B. burgdorferi* transmission and the value of this protein as a candidate for a tick antigen-based vaccine to prevent *B. burgdorferi* transmission and/or infection.

## Data availability statement

The data presented in the study are deposited in the ProteomeXchange (https://proteomecentral.proteomexchange.org/cgi/GetDataset) and MassIVE partner repository (https://massive.ucsd.edu/ProteoSAFe/datasets.jsp#%7B%22query%22%3A%7B%7D%7D), accession numbers PXD045523 and MSV000092905.

## Ethics statement

The animal study was approved by Texas A&M 102 University Institutional Animal Care and Use Committee, Animal Welfare Act, Public Health 104 Service Policy, and Humane Care and Use of Laboratory Animals. The study was conducted in accordance with the local legislation and institutional requirements.

## Author contributions

EB: Conceptualization, Data curation, Formal Analysis, Investigation, Methodology, Software, Validation, Visualization, Writing – original draft, Writing – review & editing. TK: Formal Analysis, Investigation, Methodology, Validation, Writing – review & editing. TN: Investigation, Methodology, Writing – review & editing. JB: Investigation, Writing – review & editing. JL: Validation, Writing – review & editing. LA: Conceptualization, Formal Analysis, Methodology, Software, Validation, Visualization, Writing – original draft, Writing – review & editing. LS: Methodology, Software, Writing – review & editing. SB: Methodology, Resources, Software, Writing – review & editing. DS: Formal Analysis, Investigation, Resources, Validation, Writing – review & editing. SK: Methodology, Writing – review & editing. YJ: Validation, Writing – review & editing. AM: Conceptualization, Data curation, Formal Analysis, Funding acquisition, Methodology, Project administration, Resources, Software, Supervision, Validation, Visualization, Writing – original draft, Writing – review & editing.

## Supplementary material

Supplementary Figure 1 Recombinant (r) *Ixs*S41 expressed as a glycoprotein in *Pichia pastoris*. Approximately 7 µg of **(A)** non-deglycosylated r*Ixs*S41, **(B)** deglycosylated under non-denaturing conditions, and **(C)** deglycosylated under denaturing conditions were subjected to SDS-PAGE (10%) and silver staining. Black arrows indicate bands that were excised and processed for LC-MS/MS. Identification of proteins from each band is shown in **Supplementary Table 1**.
