# Supplementary material for: Ixodes scapularis nymph saliva protein blocks host inflammation and complement-mediated killing of Lyme disease agent, Borrelia burgdorferi
Source: Front Cell Infect Microbiol. 2023 Oct 26;13:1253670. doi: 10.3389/fcimb.2023.1253670 (PMC10641286; doi:10.3389/fcimb.2023.1253670)
Supplement: Supplementary file 2 [file DataSheet_1.docx]

**Artificial Intelligence/Deep Learning Model for Quantitation of Mast Cells
in Toluidine Blue Stained Tissues**

**A. Image Processing**

Digitized images (resolution of 0.101563 μm/pixel) were uploaded to the Aiforia™ image processing and management platform (Aiforia Inc., Cambridge, MA, USA) for analysis with deep learning convolutional neural networks (CNNs) and supervised learning.

**B. AI Model Training**

*Training images:* A supervised convolutional neural network (CNN) was trained on annotations from digitized Toluidine Blue stained mouse paw sections to recognize total Mast Cell positive staining. The AI model was trained on the most diverse and representative whole-slide images (21 images for Tissue training and 14 images for Mast Cell training (35% of the total dataset)) to create a generalizable AI model capable of accurately detecting total mouse paw tissue area as well as count mast cell positive staining. Diverse training data were included to capture the variability in image/staining quality as well as slides with known artifacts (lint, bubbles, etc.) and trained the AI model to exclude them from analysis as background.

*AI model design and ground truth definitions:* The AI model consisted of multiple feature layers, with each feature layer containing unique classes that were annotated for CNN input training data. The AI model consisted of 2 feature layers: 1. Tissue annotated using semantic segmentation to distinguish the total tissue from the glass slide, and artifact and 2. Mast cells were annotated using an object detector size of 14 μm in diameter to distinguish Mast Cell positive staining from non-Mast Cells and tissue background. The Tissue and Mast Cell layers were then merged into a chained analysis pipeline, where segmentation results from the first layer were used as a clipping mask in the next layer in order to count total mast cell positive staining across each total tissue section.

*Example training annotations for tissue and mast cells:*


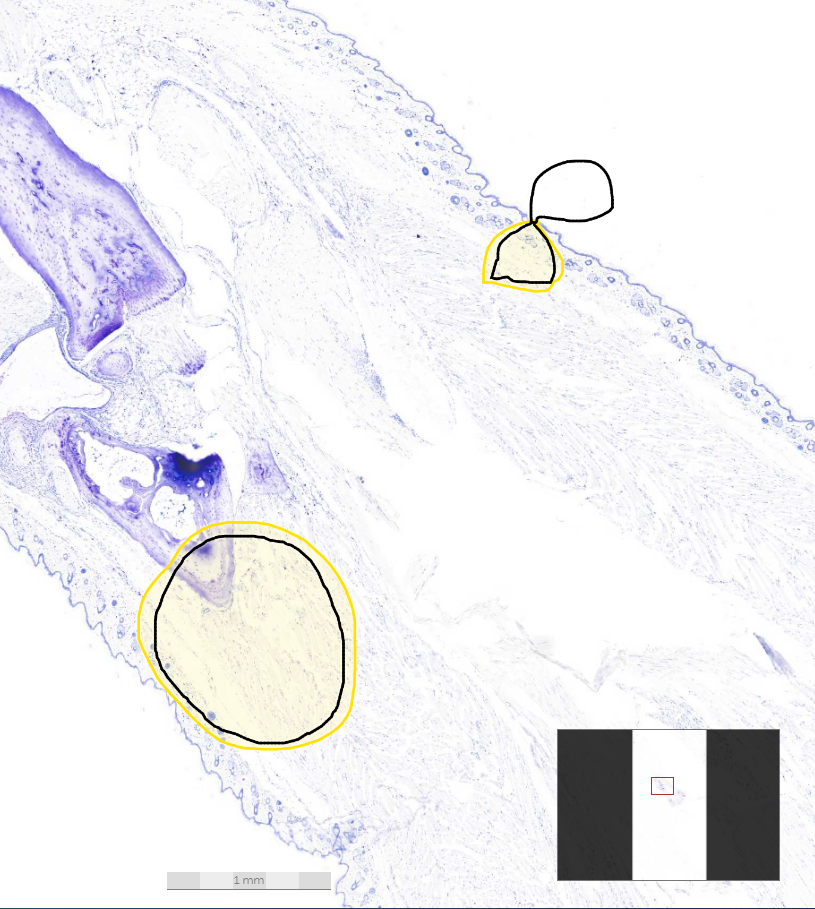

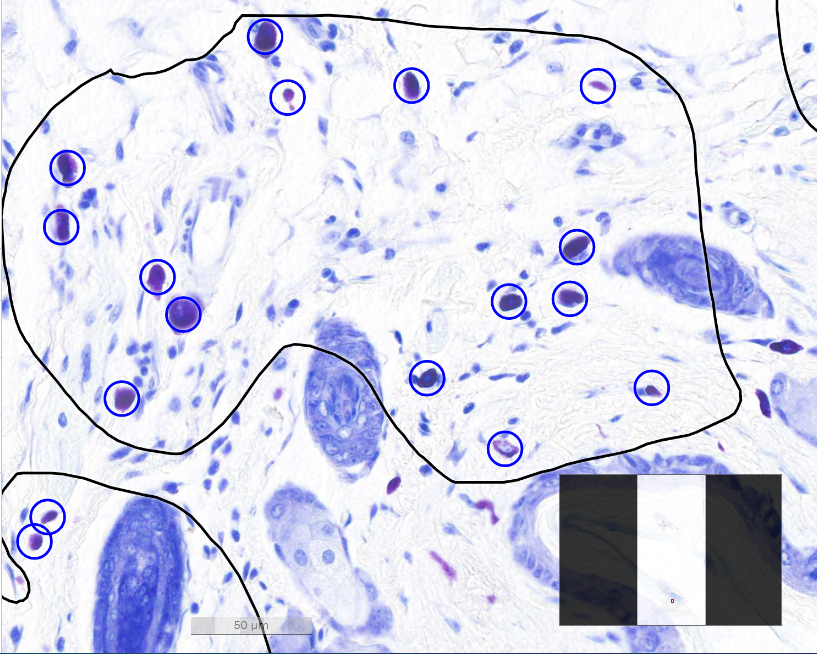


*Additional AI model training parameters:* Individual CNNs were trained for each layer using the image augmentation parameters, perceptive view (field of view), and level complexity summarized in Table 1.

**Table 1: CNN Image Augmentation Parameters, Field of View, and Complexity**

|  | **Layer 1: Tissue** | **Layer 2: Mast Cell count** |
| --- | --- | --- |
| **Scale (max/min)** | -2/2 | -10/10 |
| **Aspect Ratio** | 2 | 10 |
| **Maximum Shear** | 2 | 10 |
| **Luminance (max/min)** | -1/1 | -10/10 |
| **Contrast (max/min)** | -2/2 | -10/10 |
| **Max. white balance change** | 2 | 2 |
| **Noise** | 1 | 1 |
| **Field of View** | 800 | NA |
| **Complexity** | Extra Complex | Very Complex |

**C. AI Model Performance***Verification:* The Tissue layer was trained on approximately 118.92 mm2 of annotations (21 images total) for 10,000 iterations and the resulting AI model performed at a total verification error rate of approximately 0.08% (0.05% false positive; 0.03% false negative). The Mast Cell counter was trained on 2,567 object annotations (14 images total) for 10,000 iterations, and the resulting AI model performed at a total verification error rate of approximately 1.52% (0.86% false positive; 0.66% false negative) compared to the input training annotations. *Validation:* The AI model was tested for non-inferiority against annotations from four blinded individual external validators, board certified veterinary pathologists (BW, LS, RR, YJ) on 58 validation regions on 5 images that were separate from training tissue sections. AI performance versus the four human annotators ground truth resulted in the following error rates, precision, sensitivity, and F1-score for each layer (Table 2).

**Table 2: Error, Precision, Sensitivity, F1-score AI vs Humans**

|  | **AI vs. Human Average** | **Human vs. Human Average** |
| --- | --- | --- |
| **False Positive %** | 3.59 | 1.53 |
| **False Negative %** | 1.13 | 1.23 |
| **Total Error %** | 3.96 | 2.37 |
| **Precision %** | 97.13 | 98.77 |
| **Sensitivity %** | 98.87 | 98.77 |
| **F1-score %** | 97.78 | 98.65 |
