# Supplementary material for: Ixodes scapularis nymph saliva protein blocks host inflammation and complement-mediated killing of Lyme disease agent, Borrelia burgdorferi
Source: Front Cell Infect Microbiol. 2023 Oct 26;13:1253670. doi: 10.3389/fcimb.2023.1253670 (PMC10641286; doi:10.3389/fcimb.2023.1253670)
Supplement: Supplementary file 3 [file DataSheet_2.docx]

**Artificial Intelligence/Deep Learning Model for Quantitation of Neutrophils
in Hematoxylin and Eosin Stained Tissues**

**A. Image Processing**

Digitized images (resolution of 0.101563 μm/pixel & 0.20727 μm/pixel) were uploaded to the Aiforia™ image processing and management platform (Aiforia Inc., Cambridge, MA, USA) for analysis with deep learning convolutional neural networks (CNNs) and supervised learning.

**B. AI Model Training:**

*Training images:* A supervised convolutional neural network (CNN) was trained on annotations from digitized H&E-stained kidney, bladder, uterus, cervix and vagina sections to recognize total Neutrophil-positive staining. The algorithm was trained on the most diverse and representative 53 whole-slide images (31% of the total datasets) to create a generalizable AI model capable of accurately detecting Neutrophil-positive staining. Diverse training data were included to capture the variability in image/staining quality. We also chose slides with known artifacts (lint, bubbles etc.) and trained the AI model to exclude them from analysis as background

*AI model design and ground truth definitions:* The AI model consisted of multiple feature layers, with each feature layer containing unique classes that were annotated for CNN input training data. The AI model consisted of 2 feature layers: 1. Tissue annotated using semantic segmentation to distinguish the total tissue from the glass slide, non-kidney tissue, and artifact; 2. Neutrophils were annotated using an object detector size of 6 μm in diameter to distinguish neutrophil positive staining from non-neutrophil cells and tissue background. The Tissue and Neutrophil layers were then merged into a chained analysis pipeline, where segmentation results from the first layer (Tissue) were used as a clipping mask in the next layer (Neutrophil), and so on, to count total neutrophil positive staining across each total tissue section.

*Example training annotations for tissue and neutrophils:*


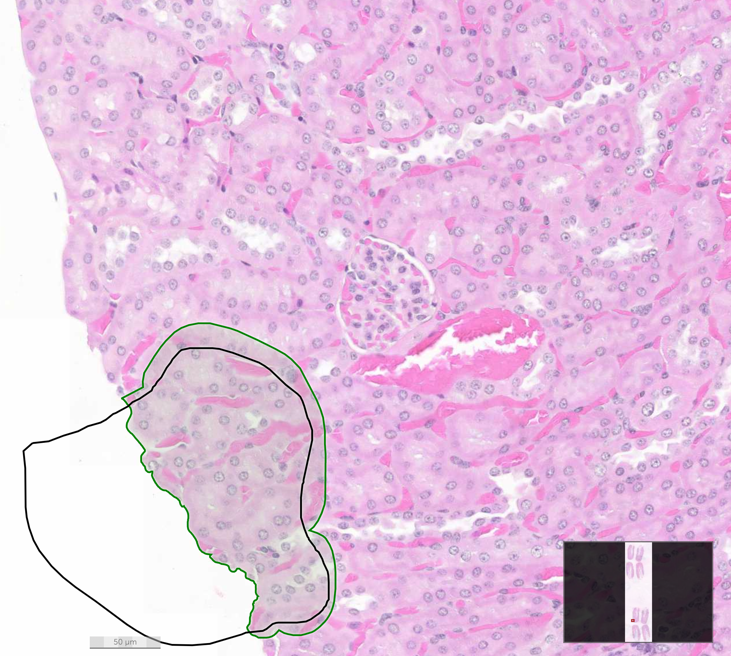

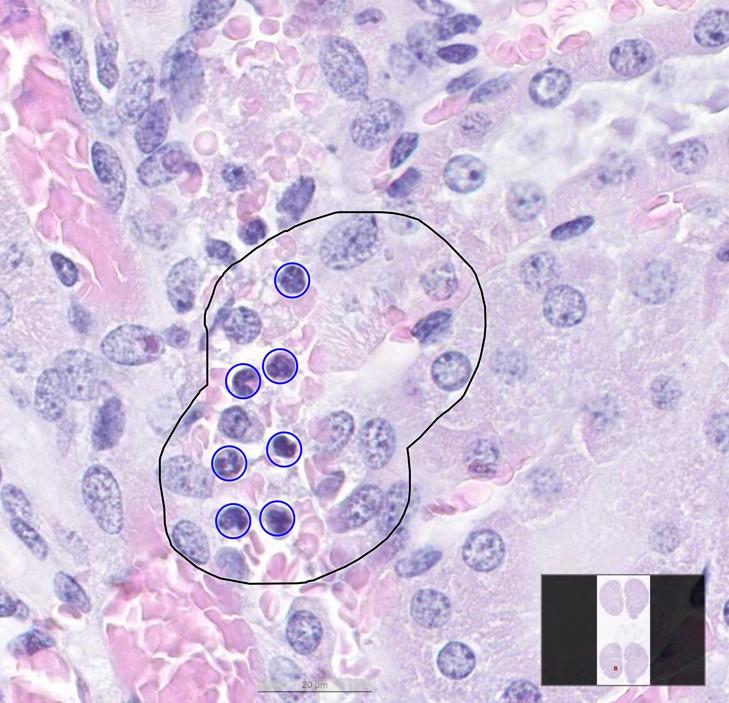


*Additional AI model training parameters:* Individual CNNs were trained for each layer using the image augmentation parameters, perceptive view (field of view), and level complexity summarized in Table 1.

**Table 1: CNN Image Augmentation Parameters, Field of View, and Complexity**

|  | **Layer 1: Tissue** | **Layer 2: Neutrophil count** |
| --- | --- | --- |
| **Scale (max/min)** | -20/20 | -1/1.01 |
| **Aspect Ratio** | 20 | 1 |
| **Maximum Shear** | 20 | 1 |
| **Luminance (max/min)** | -20/20 | -1/1.01 |
| **Contrast (max/min)** | -20/20 | -1/1.01 |
| **Max. white balance change** | 3 | 1 |
| **Noise** | 2 | 0 |
| **Field of View** | 154.8 μm | NA |
| **Complexity** | Very complex | Extra Complex |

**C. AI model performance:***Verification:* The Tissue layer was trained for 25,000 iterations on approximately 586.15 mm2 and the resulting AI model performed at a total verification error rate of approximately 0.12%. The Neutrophil layer was trained for 25,000 iterations on 2,426 object annotations and the resulting AI model performed at a total verification error rate of 3.05% compared to the input training annotations.
 *Validation:* The Neutrophil AI model was tested against annotations from 6 blinded individual board certified veterinary pathologist validators (BW, GB, LS, RR, YH, GA) for non-inferiority (58 validation regions; 12 images). Validation images included samples from bladder, kidney, uterus, cervix and vagina and represented the full spectrum of histologic changes related to the neutrophil feature variability. Inter-operator agreement was reported for both human-human and AI-human comparisons and included percentages for total error, false positive, false negative, precision, sensitivity, and F1-score. The AI-human scores were non-inferior to human-human suggesting the AI is performing to the same standard as human researchers and results are summarized in the Table 2.

**Table 2: Error, Precision, Sensitivity, F1-score AI vs. Humans**

|  | **AI vs Human** | **Human vs Human** |
| --- | --- | --- |
| **False Positive %** | 15.56 | 17.79 |
| **False Negative %** | 20.53 | 23.08 |
| **Error %** | 38.12 | 40.94 |
| **Precision %** | 75.12 | 77.40 |
| **Sensitivity %** | 79.47 | 77.40 |
| **F1-Score %** | **86.57** | **86.81** |
