# Supplementary figures and images for: Ixodes scapularis nymph saliva protein blocks host inflammation and complement-mediated killing of Lyme disease agent, Borrelia burgdorferi
Source: Front Cell Infect Microbiol. 2023 Oct 26;13:1253670. doi: 10.3389/fcimb.2023.1253670 (PMC10641286; doi:10.3389/fcimb.2023.1253670)

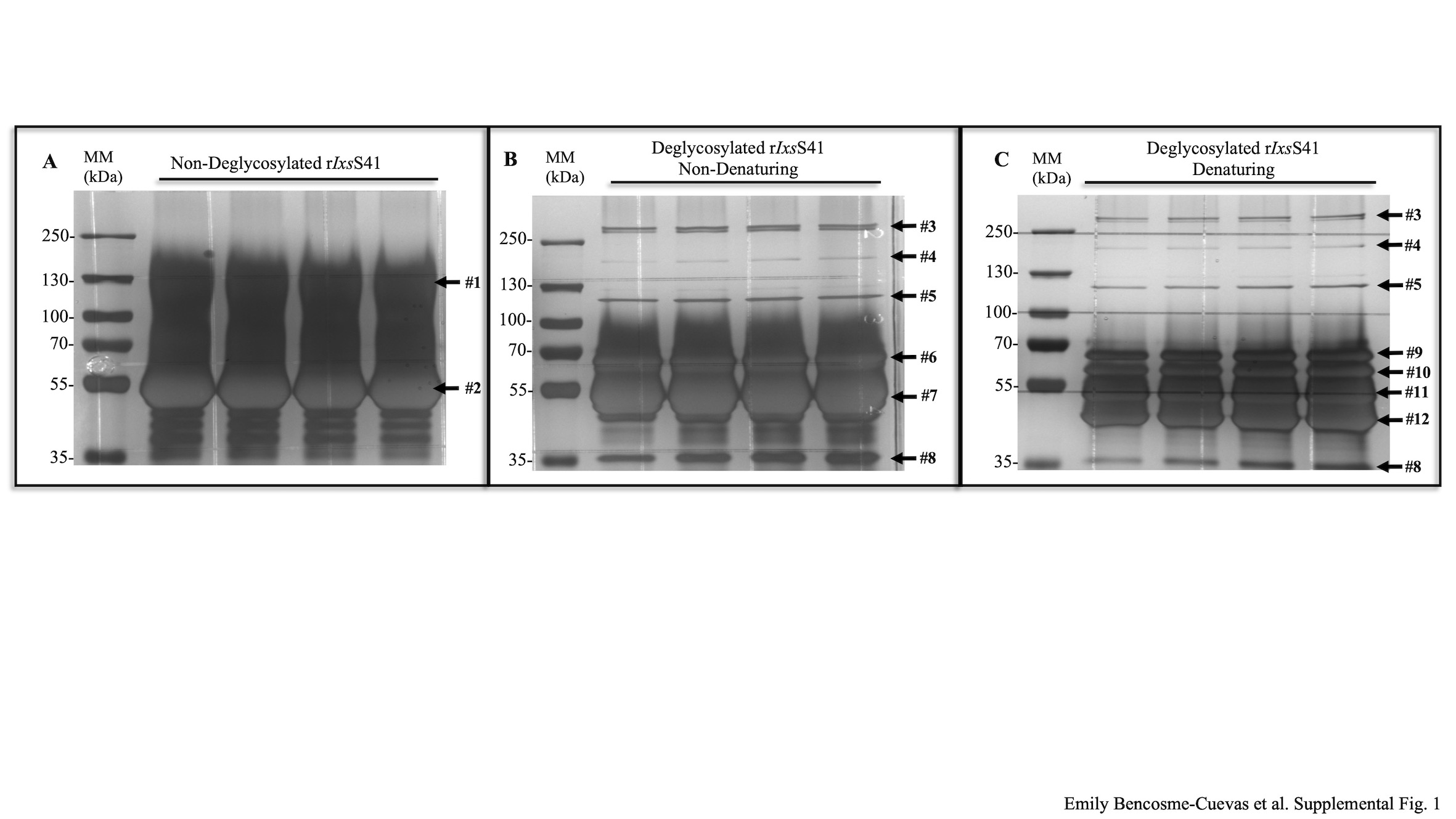

Supplement: Supplementary Figure 1 — Recombinant (r) IxsS41 expressed as a glycoprotein in Pichia pastoris. Approximately 7 µg of (A) non-deglycosylated rIxsS41, (B) deglycosylated under non-denaturing conditions, and (C) deglycosylated under denaturing conditions were subjected to SDS-PAGE (10%) and silver staining. Black arrows indicate bands that were excised and processed for LC-MS/MS. Identification of proteins from each band is shown in Supplementary Table 1 . [file Image_1.jpeg]
